# A Gene Gravity Model for the Evolution of Cancer Genomes: A Study of 3,000 Cancer Genomes across 9 Cancer Types

**DOI:** 10.1371/journal.pcbi.1004497

**Published:** 2015-09-09

**Authors:** Feixiong Cheng, Chuang Liu, Chen-Ching Lin, Junfei Zhao, Peilin Jia, Wen-Hsiung Li, Zhongming Zhao

**Affiliations:** 1 Department of Biomedical Informatics, Vanderbilt University School of Medicine, Nashville, Tennessee, United States of America; 2 Alibaba Research Center for Complexity Sciences, Hangzhou Normal University, Hangzhou, Zhejiang, China; 3 Center for Quantitative Sciences, Vanderbilt University Medical Center, Nashville, Tennessee, United States of America; 4 Department of Ecology and Evolution, University of Chicago, Chicago, Illinois, United States of America; 5 Biodiversity Research Center and Genomics Research Center, Academia Sinica, Taipei, Taiwan; 6 Department of Cancer Biology, Vanderbilt University School of Medicine, Nashville, Tennessee, United States of America; University of Southern California, UNITED STATES

## Abstract

Cancer development and progression result from somatic evolution by an accumulation of genomic alterations. The effects of those alterations on the fitness of somatic cells lead to evolutionary adaptations such as increased cell proliferation, angiogenesis, and altered anticancer drug responses. However, there are few general mathematical models to quantitatively examine how perturbations of a single gene shape subsequent evolution of the cancer genome. In this study, we proposed the gene gravity model to study the evolution of cancer genomes by incorporating the genome-wide transcription and somatic mutation profiles of ~3,000 tumors across 9 cancer types from The Cancer Genome Atlas into a broad gene network. We found that somatic mutations of a cancer driver gene may drive cancer genome evolution by inducing mutations in other genes. This functional consequence is often generated by the combined effect of genetic and epigenetic (e.g., chromatin regulation) alterations. By quantifying cancer genome evolution using the gene gravity model, we identified six putative cancer genes (*AHNAK*, *COL11A1*, *DDX3X*, *FAT4*, *STAG2*, and *SYNE1*). The tumor genomes harboring the nonsynonymous somatic mutations in these genes had a higher mutation density at the genome level compared to the wild-type groups. Furthermore, we provided statistical evidence that hypermutation of cancer driver genes on inactive X chromosomes is a general feature in female cancer genomes. In summary, this study sheds light on the functional consequences and evolutionary characteristics of somatic mutations during tumorigenesis by propelling adaptive cancer genome evolution, which would provide new perspectives for cancer research and therapeutics.

## Author Summary

Cancer genome instabilities, such as chromosomal instability and microsatellite instability, have been recognized as a hallmark of cancer for several decades. However, distinguishing cancer functional somatic mutations from massive passenger mutations and non-genetic events is a major challenge in cancer research. Massive genomic alterations present researchers with a dilemma: does this somatic genome evolution contribute to cancer, or is it simply a byproduct of cellular processes gone awry? In this study, we developed a new mathematical model to incorporate the genome-wide transcription and somatic mutation profiles of ~3,000 tumors across 9 cancer types from The Cancer Genome Atlas into a broad gene network. We found that cancer driver genes may shape somatic genome evolution by inducing mutations in other genes in cancer. This functional consequence is often generated by the combined effect of genetic and epigenetic alterations (e.g. chromatin regulation). Moreover, we provided statistical evidence that hypermutation of cancer driver genes on inactive X chromosomes is a general feature in female cancer genomes and found a putative X-inactive specific gene *STAG2* in uterine cancer. In summary, this work illustrates the functional consequences and evolutionary characteristics of somatic mutations during tumorigenesis through driving adaptive cancer genome evolution.

## Introduction

Cancer development and progression are mediated by the accumulation of genomic alterations, including point mutations, insertions and deletions, gene fusions, amplifications, and chromosomal rearrangements [1,2]. The majority of the somatic mutations found in tumor cells are ‘passenger’ rather than ‘driver’ mutations [3]. In 1976, Peter Nowell wrote a landmark perspective for the clonal evolution model of cancer and applied evolutionary models to understand tumor growth and treatment failure [4]. He proposed that most neoplasms arise from a single cell, and tumor progression results from acquired genetic variability within the original clone, allowing sequential selection of more aggressive sublines. He also noted that genetic instability, occurring in tumor cells during disease progression, might enhance this process. This view now has been widely accepted [4,5]. Somatic cell evolution leads to adaptive cancer cell survival, including increased proliferative, angiogenic, and invasive phenotypes [2]. However, understanding how somatic cell evolution drives tumorigenesis remains a great challenge in cancer research.

Genome instabilities, such as chromosomal instability and microsatellite instability, have been well studied in cellular systems [2,6,7]. For example, Teng et al. found that in yeast a mutation on a single gene may cause genomic instability, leading to adaptive genetic changes [8]. Whether and how human tumor genomes are genetically unstable, induced by single gene alterations, has been debated for decades [9–12], but has recently gained much support. For instance, Emerling et al. found an amplification of *PIP4K2B* in *HER-2/Neu*-positive breast cancer with its co-occurrence with mutations in *TP53* [11]. They showed that a subset of breast cancer patients had a high level of gene expression of *PIP4K2A* and *PIP4K2B* and provided evidence that these kinases are essential for growth in the absence of p53. Liu et al. found that *POLR2A* (encoding the largest and catalytic subunit of the RNA polymerase II complex) was deleted together with *TP53* in cancer cell lines and primary tumors in human colon cancer [13]. Additionally, the DNA cytidine deaminase APOBEC3B-catalyzed genomic uracil lesions are responsible for a large proportion of both dispersed and clustered mutations in multiple distinct cancers [12]. These lines of evidence show that single gene alterations may induce the mutations of other genes in a cancer genome that drive tumorigenesis and tumor progression [9–13]. Thus, a quantitative assessment of whether the perturbation of any single gene in a cancer genome is sufficient to drive genetic changes would help us better understand tumorigenesis and tumor evolution through genomic alterations. However, distinguishing functional somatic mutations from massive passenger mutations and non-genetic events is a major challenge in cancer research. Massive genomic alterations present researchers with a dilemma: does this somatic genome evolution contribute to cancer, or is it simply a byproduct of cellular processes gone awry [14]?

Cells consist of various molecular structures that form complex, dynamic, and plastic networks [15]. In the molecular network framework, a genetic aberration may cause network architectural changes through affecting or removing a node or its connection within the network, or changing the biochemical properties of a node (protein) [16–18]. The abundance of next-generation sequencing data of cancer genomes provides biologists with an unprecedented opportunity to gain a network-level understanding of tumorigenesis and tumor progression [15,19–22]. However, how to integrate large-scale molecular networks with cancer genomic aberrations is highly challenging [9,10]. The development of a mathematical model will be helpful to understand how genetic aberrations perturb the molecular network architecture and manifest the effects during tumorigenesis.

In this study, we proposed a novel mathematical model, namely gene gravity model, derived from Newton’s law of gravitation to study the evolution of cancer genomes. The gene gravity model detects a gene-gene pair that two genes are co-mutated and highly co-expressed simultaneously in a given cancer type based on several previous evidences [8,11,13]. As proof of principle, we applied the model to approximately 3,000 tumors’ transcription and somatic mutation profiles across 9 cancer types from The Cancer Genome Atlas (TCGA) project. We found that cancer driver genes may shape somatic genome evolution by inducing mutations in other genes during tumorigenesis. We identified six putative cancer genes by quantifying the gene gravity model. Furthermore, we found a higher somatic mutation density related to cancer driver genes on the X chromosome in comparison to the whole autosomes, suggesting that hypermutation in inactive X chromosomes is a general feature in females. In summary, this study would provide new insights into adaptive cancer genome evolution shaped by somatic mutations in cancer.

## Results

### Overview of the gene gravity model

**Fig 1:**
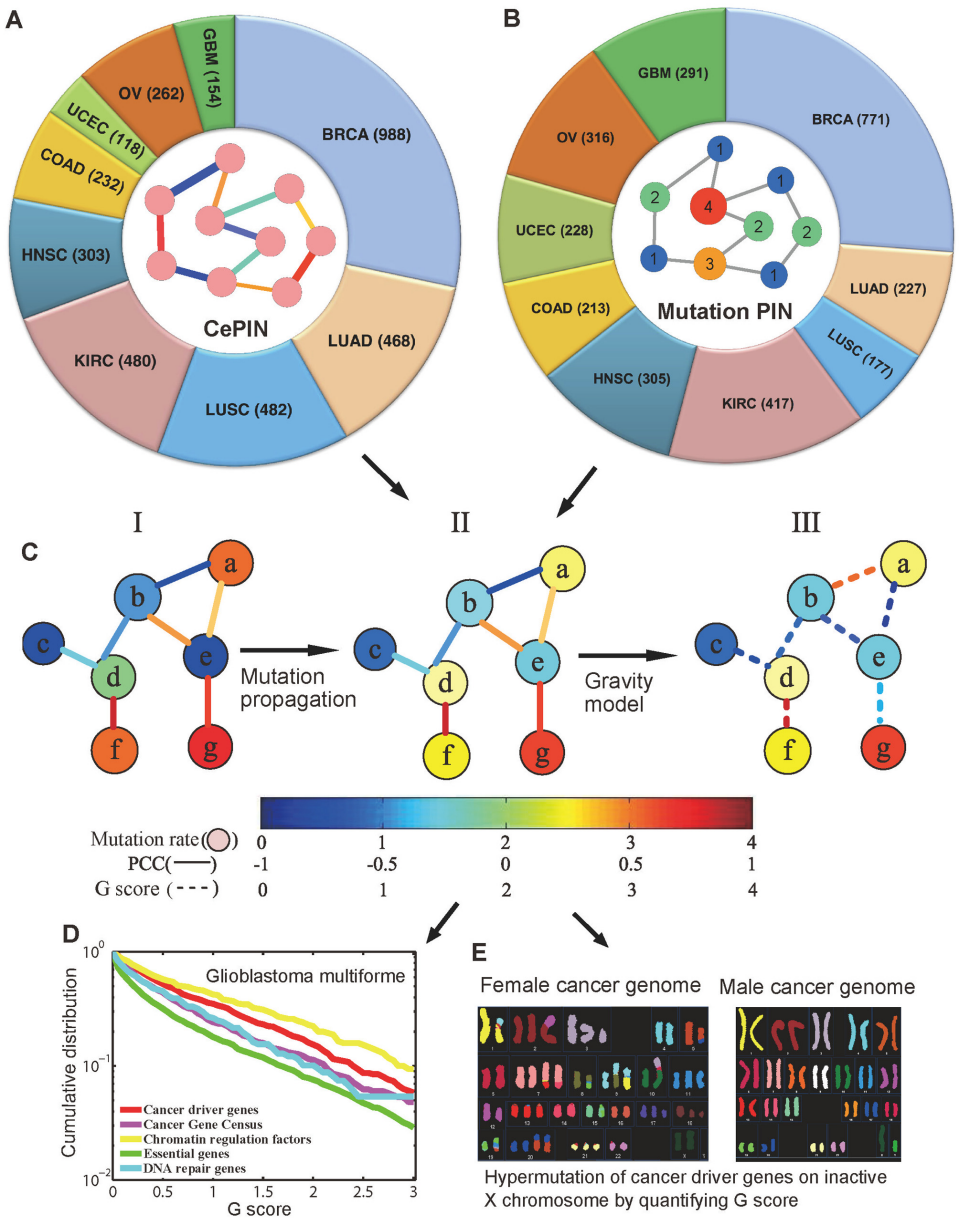
Diagram of a gene gravity model and its application to pan-cancer analysis. The gene gravity model postulates that if two genes had high mutation rates and strong gene co-expression in a given cancer type, they would exhibit a higher gravitation score (*G*) and create a higher risk of inducing mutations to other genes. (**A)** Construction of co-expressed protein interaction network (CePIN) using tumor transcription profiles from 3,487 tumors across 9 cancer types (S1 Table). (**B)** Construction of somatic mutation protein interaction network (mutation PIN) by incorporating somatic mutation profiles from 2,946 tumors across 9 cancer types in a large-scale protein interaction network. (**C)** Gene gravity model diagram. First, we used the network propagation algorithm to propagate the somatic mutations from each cancer type into PIN (I to II). We then calculated the gene-gene gravitational interaction by incorporating CePIN and mutation PIN (II to III). (**D**) Quantitatively measuring the genomic instability risk using the gravitation (*G*) score for five gene sets: Cancer driver genes, Cancer Gene Census, Chromatin regulation factors, Essential genes, and DNA repair genes, using glioblastoma multiforme (GBM) as an example. (**E**) Hypermutation of the cancer driver genes on the inactive X chromosome versus all autosomes in the female cancer genomes based on the *G* score. BRCA: breast invasive carcinoma, COAD: colon adenocarcinoma, HNSC: head and neck squamous cell carcinoma, KIRC: kidney renal clear cell carcinoma, LUAD: lung adenocarcinoma, LUSC: lung squamous cell carcinoma, OV: ovarian serous cystadenocarcinoma, and UCEC: uterine corpus endometrial carcinoma.

The gene gravity model postulates that if two genes have high mutation density and strong gene co-expression in a given cancer type, they should have a higher *G* score and related to a higher risk of inducing mutations to other genes; this postulation is based on several previous observations [8,11,13]. We developed the gene gravity model by incorporating ~3,000 tumors’ transcription and somatic mutation profiles across 9 cancer types from TCGA under molecular network architecture knowledge (Fig 1). These 9 cancer types consist of breast invasive carcinoma (BRCA), colon adenocarcinoma (COAD), glioblastoma multiforme (GBM), head and neck squamous cell carcinoma (HNSC), kidney renal clear cell carcinoma (KIRC), lung adenocarcinoma (LUAD), lung squamous cell carcinoma (LUSC), ovarian serous cystadenocarcinoma (OV), and uterine corpus endometrial carcinoma (UCEC). First, we collected 3,487 tumor transcription profiles (RNA-Seq) for the 9 cancer types. Then, we constructed 9 co-expressed protein interaction networks (CePINs) for the 9 cancer types (S1 Table) respectively by incorporating the transcription profiles into a large-scale protein interaction network (PIN) in S2 Table and Fig 1A. Each CePIN contained ~100,000 edges connecting ~12,000 genes. Second, we collected 277,370 nonsynonymous somatic mutations identified from 2,946 tumor exomes across 9 cancer types from TCGA (S1 Table). For each cancer type, we projected the somatic mutations onto PIN to construct a somatic mutation PIN via a network propagation algorithm (Fig 1B and 1C). We then derived a *G* score for each gene-gene pair in the 9 cancer types, using Newton’s law of gravitation (Fig 1C). Then, we examined the *G* score for seven gene sets: cancer driver genes, cancer gene census (CGC) genes (experimentally validated cancer genes), tumor suppressor genes (TSGs), oncogenes, DNA repair genes, chromatin regulation factors (CRFs), and essential genes (Fig 1D). Finally, we investigated the pattern of hypermutation of the inactive X chromosome in female versus male cancer genomes by quantifying cancer genome evolution using the gene gravity model (Fig 1E).

### Benchmark evaluation of the gene gravity model

To verify the gene gravity model, we investigated the enrichment of somatic mutations on protein-protein interaction (PPI) pairs as well as unfiltered interactions relative to the same number of random pairs based a previous study [23]. We found that PIN is significantly more enriched for high mutation density than random pairs across the 9 cancer types (*q* < 2.2 × 10^−16^, Wilcoxon rank-sum test corrected by Benjamini-Hochberg multiple testing, S1 Fig). We first examined the distribution of *G* score for two benchmark gene sets: DNA repair genes and CRFs. The CRFs modulating the epigenetic landscape have emerged as potential gatekeepers and signaling coordinators for the maintenance of genome integrity [24]. The enzymes encoded by DNA repair genes continuously monitor chromosomes to repair damaged nucleotide residues generated by exposure to carcinogens and cytotoxic agents (e.g., anticancer drugs) [25]. Thus, both CRFs and DNA repair genes are of critical importance for the maintenance of the genetic information in the cancer genome. In this study, we collected two high-quality gene sets: 153 DNA repair genes [26] and 176 CRFs [27] (S3 Table). We defined a DNA repair gene-gene pair gravitational interaction as one or two genes in a pair is/are DNA repair genes. A non-DNA repair gene-gene pair gravitational interaction was defined as neither of the two genes in a pair is a DNA repair gene. We applied the same definition for the remaining 6 gene sets: cancer driver genes, CGC genes, TSGs, oncogenes, CRFs, and essential genes. We then investigated the complementary cumulative *G* score (S2–S10 Figs). We found that the DNA repair gene cumulative *G* score is higher than that of non-DNA repair genes in 8 cancer types, except BRCA. Furthermore, the CRF cumulative *G* score is higher than that of non-CRFs in all of the 9 cancer types (S2–S10 Figs). Collectively, these observations demonstrated that we could use the gene gravity model to quantitatively examine how perturbations of a single gene shape subsequent evolution of the cancer genome based on evidence in several previous biological studies [8,11,13].

### High somatic evolutionary pressure for the mutated cancer driver genes

We investigated “high somatic evolutionary pressure” for a particular gene that tends to be co-mutated and highly co-expressed with other genes in a given cancer type. We hypothesized that if a gene has a higher somatic evolutionary pressure, this gene may increase subsequent genetic changes [8,11,13]. We compiled a high-quality, mutated cancer driver gene set (614 cancer driver genes, S3 Table) from four pan-cancer genomic analysis projects [3,28–30]. We found that the cancer driver gene cumulative *G* score is significantly higher than that of non-cancer driver genes in all of the 9 cancer types (*q* < 2.2 × 10^−16^, Wilcoxon rank-sum test, S2–S10 Figs). These observations suggest that cancer driver mutations may increase subsequent genetic changes based on the previous studies [8,11,13]. We also studied CGC genes, which are well curated and have been widely used as a reference cancer gene set in many cancer-related studies [31,32]. As expected, we found that the CGC gene cumulative *G* score is higher than that of non-CGC genes in 6 cancer types: BRCA, COAD, GBM, HNSC, KIRC, and UCEC (S2–S6 and S10 Figs).

However, the CGC gene cumulative *G* score is slightly higher than that of non-CGC genes in 3 cancer types: LUAD, LUSC, and OV (S7–S9 Figs). A previous study indicated that an average mutation frequency in smokers is more than 10-fold higher in never-smokers in non-small cell lung cancer [33]. We next separated TCGA patients into smokers and never-smokers in LUAD and LUSC, and reexamined the CGC gene cumulative *G* score. As expected, the CGC gene cumulative *G* score is significantly higher than that of non-CGC genes in LUAD and LUSC never-smokers (*q* < 0.05, S11 Fig). However, the CGC gene cumulative *G* score is slightly higher than that of non-CGC genes in LUAD and LUSC smokers (S11 Fig). Thus, heterogeneous mutation frequencies and gene transcription profiles in the combined smokers and never-smokers in LUAD or LUSC may influence the performance of the gene gravity model [33]. For OV (S9 Fig), high genomic instability of the ovarian cancer genome may cause this slight gene cumulative *G* score between CGC and non-CGC genes [34]. Finally, we considered essential genes. We compiled 2,719 essential genes (S3 Table) from the Online GEne Essentiality database [35]. S2–S10 Figs showed that the essential gene cumulative *G* score is higher than that of non-essential genes across 9 cancer types. Remarkably, the cancer driver gene-gene *G* score is higher than that of essential genes (*q* < 0.01) in all of the 9 cancer types (S2–S10 Figs).

Tumorigenesis is dependent on the accumulation of one or multiple driver mutations that activate oncogenic pathways or inactivate tumor suppressors [36,37]. Oncogenes often positively co-expressed with interacting partners due to gain-of-function mutations; while TSGs often negatively co-expressed with interacting partners due to lose-of-function mutations [38]. Thus, we defined attractive gravitation (*AG*) as two genes that have positive gene co-expressed correlation and repulsive gravitation (*RG*) as two genes that have negative gene co-expressed correlation in a specific cancer type. We compiled 477 oncogenes and 1,040 TSGs (S3 Table), and then examined the *AG* and *RG* score for oncogenes and TSGs, respectively. We found that the oncogene *AG* cumulative distribution is higher than that of non-oncogenes in 5 cancer types: BRCA, COAD, KIRC, OV, and UCEC (S12 Fig). However, as shown in S13 Fig, the oncogene *RG* cumulative distribution is similar or slightly higher than that of non-oncogenes in all of the 9 cancer types. Additionally, we examined the *AG* and *RG* score for TSGs. We found that both *AG* and *RG* cumulative distribution for TSGs is higher than that of non-TSGs in 7 cancer types, except LUSC and OV (S14 and S15 Figs). Taken together, our gene gravity model can distinguish one important tumor biological characteristics, oncogenic potential altered by oncogenes, very well. However, our model fails to distinguish caretaker or gatekeeper roles altered by TSGs. One possible reason is that some TSGs have both tumor suppressor and oncogenic activities in different cancer types or cell types. For example, p21, encoded by *CDKN1A*, plays both tumor suppressor activities and paradoxical tumor-promoting activities in cancer [39]. In addition, it is partially because TSGs have truncated mutations that may scattered in the gene region. Thus, further study will be needed for systematic investigation of the *AG* and *RG* score for TSGs, which we hope will be prompted by the findings herein.

### Combinatorial effects of the cancer evolution induced by genetic and epigenetic alterations

**Fig 2:**
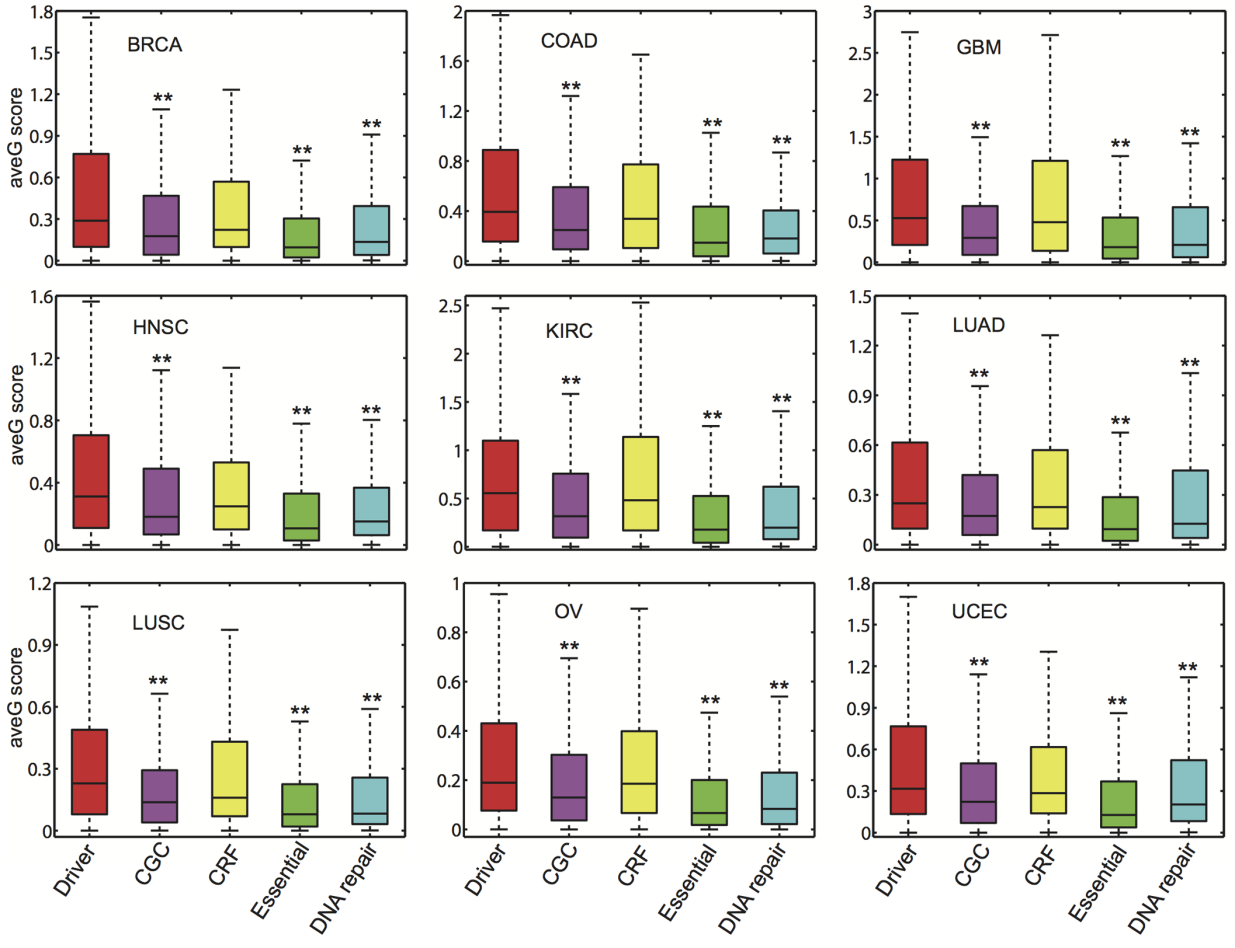
Box plots of gene average gravitation (ave*G*) score for five gene sets across 9 cancer types. Red: cancer driver genes (Driver); purple: Cancer Gene Census (CGC) genes; yellow: chromatin regulation factors (CRF); green: essential genes (Essential); and blue: DNA repair genes. The adjusted p-values (*q*) are based on the comparison of the gene average gravitation score of cancer driver genes with CGC, CRFs, essential genes, and DNA repair genes respectively, by Wilcoxon rank-sum test corrected by Benjamini-Hochberg multiple testing. **: *q* < 0.01. The detailed data are provided in S4 Table. Abbreviations of 9 cancer types in Figs 2–5 are provided in Fig 1 legend.

We calculated the gene average gravitation (ave*G*) score using (*ρ*)_*i*_ = ∑_*j*_ *G*_*ij*_ / *n* between gene *i* and gene *j* (*j* belongs to the set of gene *i*’s interacting partners (n) in PIN). We found that the ave*G* score of cancer driver gene is significantly higher than that of DNA repair, CGC, and essential genes in all of the 9 cancer types (Fig 2 and S4 Table). For BRCA, the cancer driver gene ave*G* score (0.47 ± 0.02) is significantly higher than that of DNA repair genes (0.30 ± 0.03, *q* = 1.9 × 10^−4^), CGC genes (0.35 ± 0.02, *q* = 1.1 × 10^−4^), and essential genes (0.26 ± 0.01, *q* = 2.3 × 10^−32^, S4 Table). However, the cancer driver gene ave*G* score is similar to that of CRFs (0.42 ± 0.04, *q* = 1.0) in BRCA. Similar trends were observed in the remaining 8 cancer types (S4 Table). Thus, chromatin regulation might play an important role in tumorigenesis.

**Fig 3:**
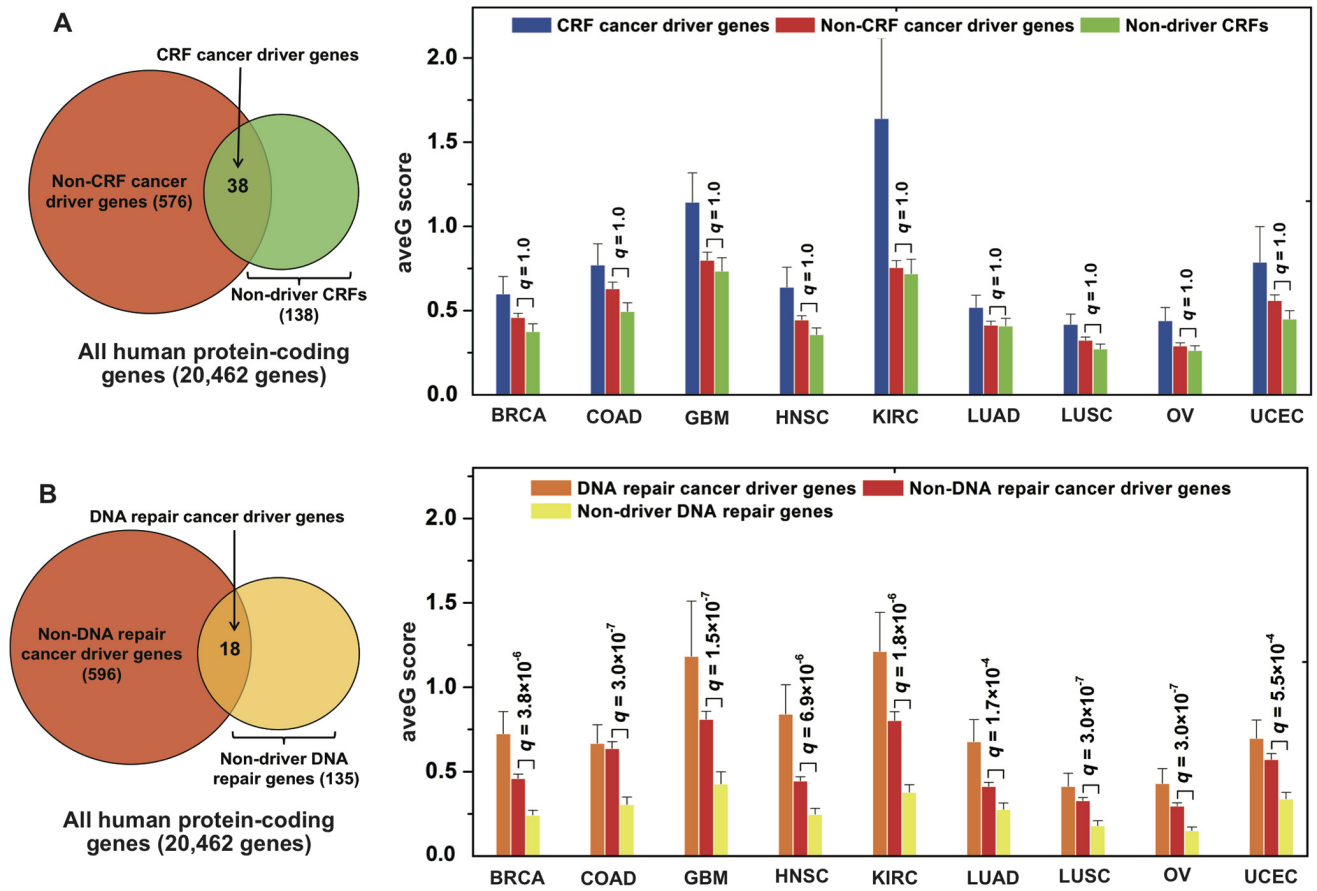
Combined effects of genetic and epigenetic alterations. The left Venn diagrams show the relationship between cancer driver genes and (**A**) chromatin regulation factors (CRFs) and (**B**) DNA repair genes. The right panels show the distributions of the average gravitation (ave*G*) score of three gene sets in the corresponding left Venn diagram across 9 cancer types: (**A**) comparison of CRF cancer driver genes, non-CRF cancer driver genes, and non-driver CRF genes; (**B**) comparison of DNA repair cancer driver genes, non-DNA repair cancer driver genes, and non-driver DNA repair genes. The adjusted p-values (*q*) are calculated by the Wilcoxon rank-sum test and corrected by Benjamini-Hochberg multiple testing. The detailed data is provided in S5 and S7 and S8 Tables.

We further investigated whether genetic or epigenetic alterations have combinatorial effects that shape cancer genome evolution. Since CRFs represent the epigenetic landscape [27], we divided cancer driver genes into two subgroups: CRF cancer driver genes and non-CRF cancer driver genes. We found cancer driver genes are significantly enriched in CRFs (38 out 176 CRFs versus 176 CRFs from 20,462 human protein-coding genes collected from National Center for Biotechnology Information [NCBI] database, p = 3.0 × 10^−21^, Fisher’s exact test, Fig 3A). Furthermore, the CRF cancer driver gene ave*G* score is higher than that of non-driver CRFs across 9 cancer types (*q* < 0.10, Fig 3A and S5 Table). For KIRC, the CRF cancer driver gene ave*G* score (1.6 ± 0.48) is significantly higher than that of non-CRF cancer driver genes (0.76 ± 0.04, *q* = 4.2 × 10^−3^) and non-driver CRFs (0.72 ± 0.09, *q* = 3.3 × 10^−3^, S5 Table), respectively. However, we did not find a significant ave*G* difference between non-CRF cancer driver genes and non-driver CRFs in any of the 9 cancer types (*q* = 1.0, Fig 3A and S5 Table).

We next divided CGC genes into two subgroups: CRF CGC genes and non-CRF CGC genes. We found that CGC genes are significantly enriched in CRFs as well (p = 1.2 × 10^−15^, Fisher’s exact test, S16A Fig). As expected, we did not observe a significant ave*G* difference between non-CRF CGC genes and non-CGC CRFs in 7 cancer types (*q* > 0.05, S6 Table), with the exception of OV (*q* = 0.04) and KIRC (*q* = 0.04). Put together, the cancer genome evolution might be shaped by the combinatorial synergy between cancer driver genes and CRFs.

We next divided cancer driver genes into two subgroups: DNA repair cancer driver genes and non-DNA repair cancer driver genes. Fig 3B showed that DNA repair genes tend to be cancer driver genes as well (18 out 153 DNA repair genes versus 153 DNA repair genes from 20,462 human protein-coding genes collected from NCBI database, p = 1.1 × 10^−6^). However, CRFs are more likely to be cancer driver genes than DNA repair genes (p = 0.02). The DNA repair cancer driver gene ave*G* score is similar to that of non-DNA repair cancer driver genes in 6 cancer types (*q* > 0.1), except of HNSC (*q* = 0.02, S7 Table), KIRC (*q* = 0.08), and LUAD (*q* = 0.08). However, the DNA repair cancer driver gene ave*G* score is significantly higher than that of non-driver DNA repair genes (*q* < 0.01, S7 Table) in all of the 9 cancer types (Fig 3B). For BRCA, the DNA repair cancer driver gene ave*G* score (0.73 ± 0.13) is marginally higher than that of non-DNA repair cancer driver genes (0.46 ± 0.02, *q* = 0.12), while significantly higher than that of non-driver DNA repair genes (0.24 ± 0.03, *q* = 4.4 × 10^−4^, S7 Table). Furthermore, the non-DNA repair cancer driver gene ave*G* score is significantly higher than that of non-driver DNA repair genes in all of the 9 cancer types as well (*q* < 0.01, Fig 3B and S7 Table). We further divided CGC genes into two subgroups: DNA repair CGC genes and non-DNA repair CGC genes. We found CGC genes are significantly enriched in DNA repair genes as well (p = 2.7 × 10^−18^, Fisher’s exact test, S16B Fig). S8 Table indicated that DNA repair CGC gene ave*G* score is not significantly higher than that in both non-DNA repair CGC genes (*q* > 0.50) and non-CGC DNA repair genes (*q* > 0.10) in 8 cancer types with an exception of OV (*q* = 0.03). Moreover, the non-DNA repair CGC gene ave*G* score is higher than that of non-CGC DNA repair genes in COAD (*q* = 0.04) and OV (*q* = 0.02, S8 Table). Collectively, the cancer genome evolution shaped by cancer driver genes may have additional mechanisms (i.e., chromatin regulation), except DNA repair.

### Identifying putative cancer genes by the gene gravity model

**Fig 4:**
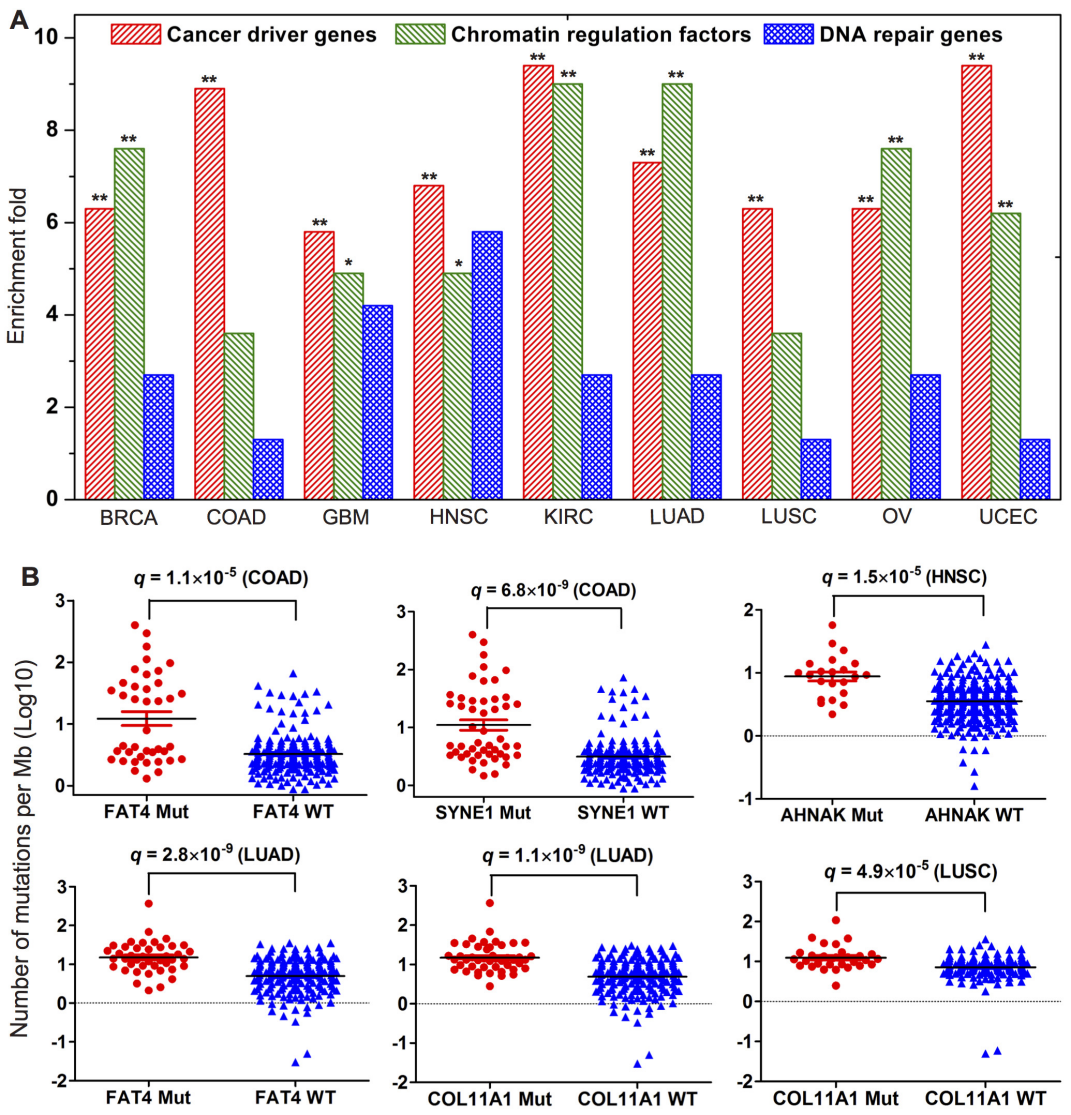
Identifying four putative cancer genes. (**A**) Enrichment analysis of the top 100 genes with the highest gene average gravitation scores for cancer driver genes, chromatin regulation factors, and DNA repairs genes. The adjusted p-values (*q*) are calculated by the Fisher’s exact test and corrected by Benjamini-Hochberg multiple testing. **: *q* < 0.01, *: *q* < 0.05. The detailed data is provided in S9 and S11 and S12 Tables. (**B**) Distribution of the number of mutations per megabase pairs (Mb) for the mutated (Mut) tumor samples versus wild-type (WT) samples. The *q* values are calculated by the Wilcoxon rank-sum test and corrected by Benjamini-Hochberg multiple testing.

**Fig 5:**
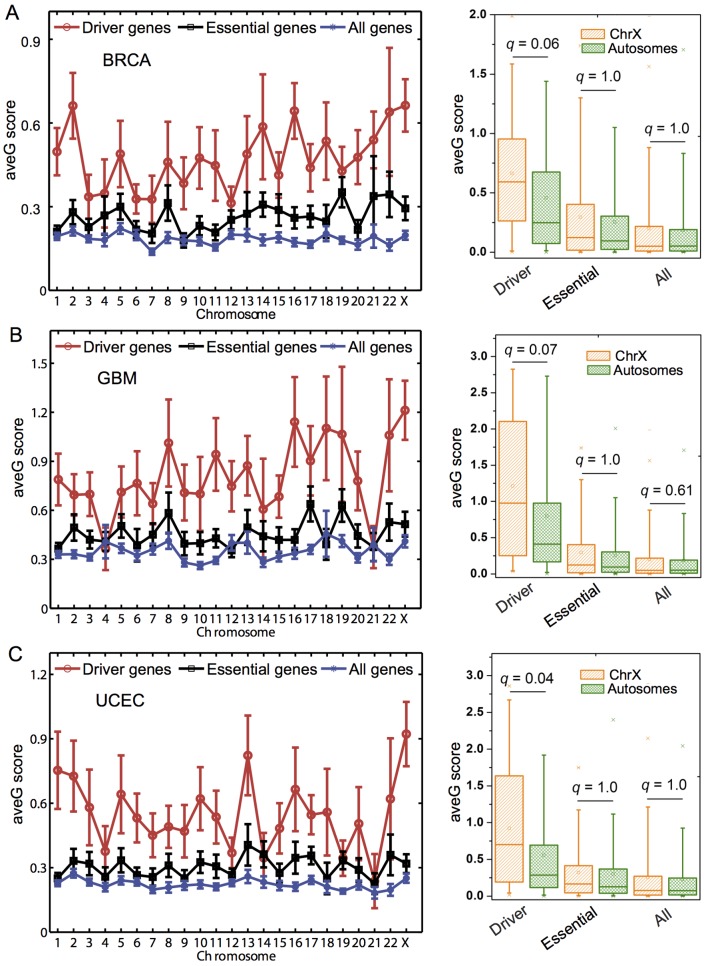
Distribution of average gravitation (ave*G*) score for cancer driver genes (Driver), essential genes (Essential), and all genes (All) across 23 human chromosomes in 3 cancer types. The left plots show the distribution of ave*G* scores for three different genes across 23 human chromosomes in (**A**) BRCA: breast invasive carcinoma, (**B**) GBM: glioblastoma multiforme, and (**C**) UCEC: uterine corpus endometrial carcinoma. The right box plots show the comparison of ave*G* scores between the X chromosome (ChrX) and all the 22 autosomes (Autosomes) for three different gene sets in the corresponding left three cancer types. The p-values are calculated by the Wilcoxon rank-sum test and corrected by Benjamini-Hochberg multiple testing. The remaining 6 cancer types are provided in S17 Fig.

We found that the top 100 genes with the highest ave*G* scores tend to be cancer driver genes (*q* < 0.01, Fisher’s exact test, Fig 4A and S9 Table) or CGC genes (*q* < 0.05, S10 Table) in all of the 9 cancer types. In addition, the top 100 genes with the highest ave*G* scores are more likely to be CRFs (*q* < 0.05, S11 Table) in 7 cancer types with the exception of COAD (*q* = 0.12) and LUSC (*q* = 0.12). However, the top 100 genes are not significantly enriched in DNA repair genes in all of the 9 cancer types (*q* > 0.05, Fig 4A and S12 Table). We further examined the tumor exome mutation density (the average number of mutations per Mb) for the top 10 genes with the highest ave*G* score via the genome-wide mutation rate analysis (S13 Table). By examining mutation density data of ~3,000 tumor exomes from Kandoth et al. [29], we found that patients having nonsynonymous somatic mutations on any of four genes (*FAT4*, *SYNE1*, *AHNAK*, or *COL11A1*) often showed a higher cancer genome mutation density at the whole genome level compared to that of wild-type (WT) patients in 4 cancer types: COAD, LUAD, LUSC, and UCEC (Fig 4B). FAT4 (protocadherin fat 4), a member of the cadherin super-family, is a key component in the Hippo signaling pathway, playing a candidate tumor suppressor role in cancer [40]. In COAD, 40 patients harbored *FAT4* nonsynonymous mutations. The average number of mutations per Mb for 40 *FAT4* mutated COAD samples (43.3 ± 12.8) are significantly higher than that of *FAT4* WT samples (5.0 ± 0.57, *q* = 1.1 × 10^−5^, Fig 4B). Similarly, the average number of mutations per Mb for 43 *FAT4* mutated LUAD samples (26.3 ± 8.4) are significantly higher than that of *FAT4* WT samples (7.5 ± 0.48, *q* = 2.8 × 10^−9^, Fig 4B). Using genome-wide association studies, Berndt et al. found *FAT4* to be a candidate gene for spontaneous pulmonary adenomas [41]. Using exome sequencing, Zang et al. found that the somatic inactivation of *FAT4* might be a critical tumorigenic event in a subset of gastric cancers [42]. In this study, *FAT4* was identified as a putative cancer gene involved in lung and colorectal cancer, which is consistent with previous studies [40–43]. *SYNE1*, encoding spectrin repeat containing, nuclear envelope 1, is involved in nuclear organization and structural integrity, function of the Golgi apparatus, and cytokinesis. Herein, we found that the average number of mutations per Mb for 49 *SYNE1* mutated COAD samples (35.8 ± 8.4) are significantly higher than that of *SYNE1* WT samples (7.5 ± 0.48, *q* = 6.8 × 10^−9^, Fig 5B). Doherty et al. found that *SYNE1* polymorphism relates to an increased risk of invasive ovarian cancer [44]. Collectively, *SYNE1* may be a candidate cancer mutated gene in COAD.

AHNAK (neuroblast differentiation-associated protein), also known as desmoyokin, is essential for tumor cell migration and invasion [45]. In this study, the average number of mutations per Mb (12.1 ± 2.6) for 22 *AHNAK* mutated samples is significantly higher than that of *AHNAK* WT samples in HNSC (4.5 ± 0.21, *q* = 1.5 × 10^−5^, Fig 4B). Dumitru et al. found that *AHNAK* was associated with poor survival rates in laryngeal carcinoma, a major subtype of head and neck cancer [46]. *COL11A1* and *COL6A3*, encoding collagen proteins, are two main structural proteins of the various connective tissues in animals. In LUAD, the average number of mutations per Mb (25.3 ± 7.9) for 46 *COL11A1* mutated samples is significantly higher than that of *COL11A1* WT samples (7.4 ± 0.47, *q* = 1.1 × 10^−9^, Fig 5B). Additionally, for LUSC, the average number of mutations per Mb (16.5 ± 0.59) for 32 *COL11A1* mutated samples is significantly higher than that of *COL11A1* WT samples as well (8.5 ± 0.40, *q* = 4.9 × 10^−5^). Furthermore, *COL6A3* (*q* = 3.1 × 10^−4^, COAD) and *COL5A2* (*q* = 1.5 × 10^−4^, LUAD) mutations are significantly associated with a high mutation density in colorectal and lung cancer, respectively. The over-expression of *COL11A1* reportedly correlates with lymph node metastasis and poor prognosis in non-small cell lung cancer and ovarian cancer [47–49]. The expression level of *COL6A3* is involved in pancreatic malignancy [50,51]. Collectively, *AHNAK*, *COL11A1*, and *COL6A3* may be potential candidates for therapeutic and diagnostic biomarkers in head and neck cancer and lung carcinoma. However, the mutation status of each of aforementioned genes is associated with the genome-wide mutation rate. Mutations in these genes could be either the cause of the mutation-rate increase or simply a consequence of an elevated global mutation rate. Thus, further experimental validation of these genes in the specific cancer type is warranted.

### Hypermutation of the inactive X chromosome in the female cancer genomes

When examining cancer driver gene ave*G* score across chromosomes in each of 9 cancer types, interestingly, we found that the X chromosome has an unusually higher cancer driver gene ave*G* scores compared to autosomes in BRCA, GBM, and UCEC using the total 22 autosomes as background (Fig 5). In BRCA, cancer driver gene ave*G* score (0.66 ± 0.09) on the X chromosome is higher than that of the whole set of 22 autosomes (0.46 ± 0.02, *q* = 0.06 [p = 7.9 × 10^−3^], Wilcoxon rank-sum test, Fig 5A). Similarly, in GBM, the cancer driver gene ave*G* score (1.2 ± 0.18) on the X chromosome is higher than that of the whole set of 22 autosomes (0.80 ± 0.05, *q* = 0.07 [p = 9.9 × 10^−3^], Fig 5B). And the cancer driver gene ave*G* score (0.92 ± 0.15) on the X chromosome is also higher than that of the whole set of 22 autosomes in UCEC (0.56 ± 0.03, *q* = 0.04 [p = 5.3 × 10^−3^], Fig 5C). As a control, we repeated the aforementioned analyses for all genes and essential genes, respectively. We did not find the higher ave*G* score on the X chromosome for all genes or essential genes in any of the 9 cancer types (Fig 5 and S17 Fig). Thus, the high gene ave*G* score on the X chromosome is unique for cancer driver genes.

**Fig 6:**
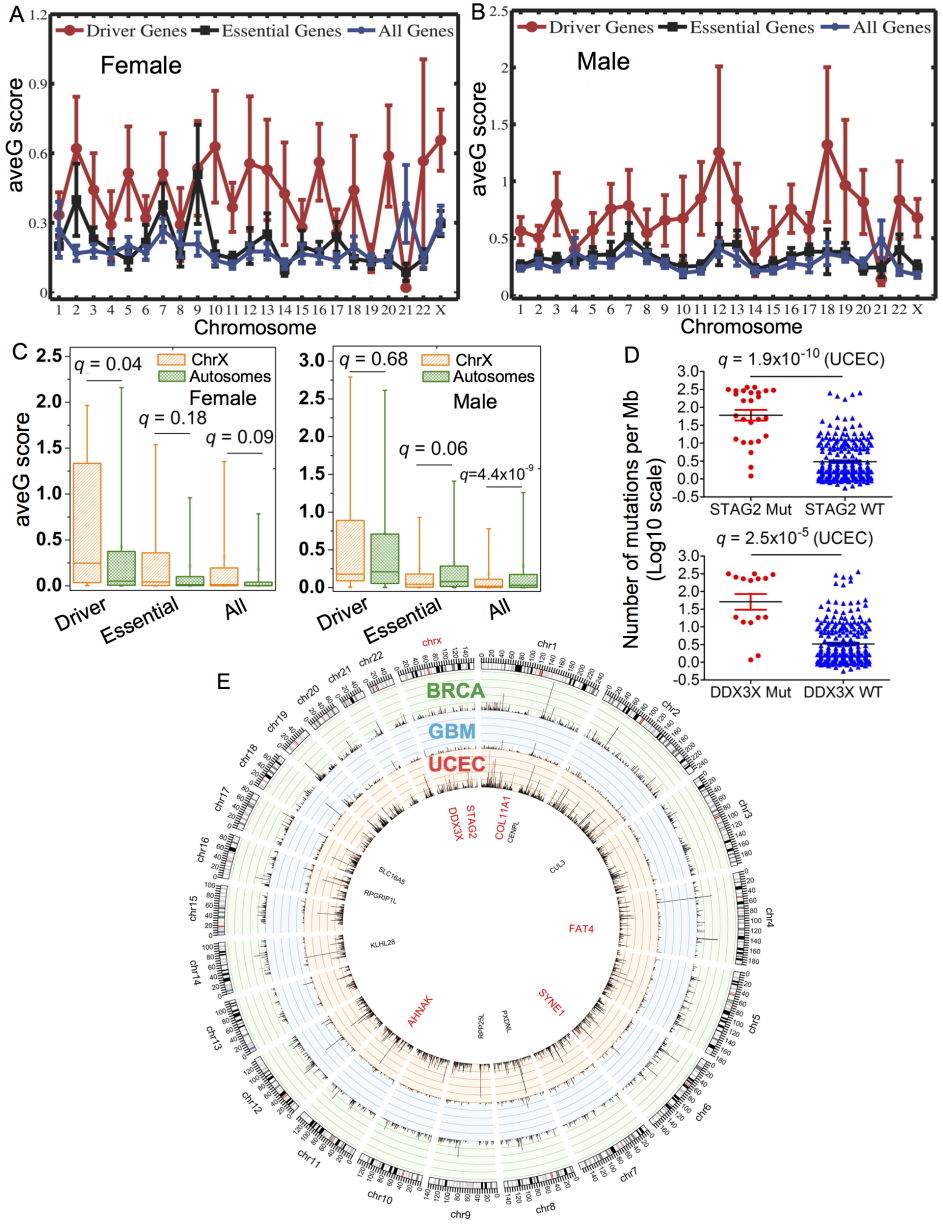
Distribution of average gravitation (ave*G*) scores for cancer driver genes (Driver), essential genes (Essential), and all genes (All) in glioblastoma multiforme (GBM) male versus female genomes across 23 human chromosomes. The distribution of ave*G* scores for (**A**) GBM female genomes and (**B**) GBM male genomes. (**C**) Box plots show the comparison of aveG scores between the X chromosome (ChrX) and all the 22 autosomes (Autosomes) for three different gene sets in GBM male versus female genomes. (**D**) Distribution of the number of mutations per megabase pairs (Mb) for the *STAG2* or *DDX3X* mutated (Mut) versus wild-type (WT) tumors in uterine corpus endometrial carcinoma. (**E**) Circos plot displaying the distribution of gene ave*G* scores for cancer driver genes (red bars) and non-cancer driver genes (black bars) across 23 human chromosomes in 3 cancers. This image is prepared by Circos (http://circos.ca). Six genes labeled in red represent the putative cancer genes identified by the gene gravity model. The p-values are calculated by the Wilcoxon rank-sum test and corrected by Benjamini-Hochberg multiple testing.

The X chromosome is largely functionally haploid in both males and females. A recent study showed that hypermutation of the inactive X chromosome is a frequent event in cancer [52]. Both BRCA and UCEC (Fig 5) are female-specific cancer, while GBM is not. To explore the hypermutation of inactive X chromosome in the female versus male cancer genomes, we separated GBM patients as males and females, and performed the same analysis. Interestingly, we found that the cancer driver gene ave*G* score (0.66 ± 0.13) on the X chromosome is significantly higher than that of the whole set of 22 autosomes (0.43 ± 0.04, *q* = 0.04, Fig 6A and 6C) in the female GBM genomes. However, the cancer driver gene ave*G* score (0.68 ± 0.17) on the X chromosome is similar to that of the whole set of 22 autosomes (0.72 ± 0.07, *q* = 0.68, Fig 6B and 6C) in the male GBM genomes. Furthermore, similar ave*G* scores for all genes (*q* = 0.09) or essential genes (*q* = 0.18) were observed between the X chromosome and the whole set of 22 autosomes in the female GBM genomes. In contrast, we found a lower ave*G* score on the X chromosome for all genes (*q* = 4.4 × 10^−9^, Fig 6C) or essential genes (*q* = 0.06) compared to that on the whole set of 22 autosomes in the male GBM genomes.

We then examined the top 10 driver genes with the highest ave*G* scores on the X chromosome in BRCA, GBM, and UCEC. Two putative cancer drivers (*DDX3X* and *STAG2*) stood out (Fig 6D and 6E). We found that the patients harboring *DDX3X* or *STAG2* nonsynonymous mutations have a higher genome mutation density in uterine cancer during the genome-wide mutation rate analysis (Fig 6D). For instance, the average number of mutations per Mb for 15 *DDX3X* mutated uterine tumors is 144.1 ± 34.0, 11-fold higher than that of *DDX3X* WT tumors (13.1 ± 2.8, *q* = 2.5 × 10^−5^). A previous study indicated that somatic mutations of *DDX3X* were associated with medulloblastoma [53]. Additionally, the average number of mutations per Mb for 26 *STAG2* mutated uterine tumors (144.5 ± 26.0) is significantly higher than that for *STAG2* WT samples (10.2 ± 2.2, *q* = 1.9 × 10^−10^). STAG2 belongs to cohesin protein family, playing an important role in mediating sister chromatid cohesion [54]. Solomon et al. found that the inactivation of *STAG2* causes aneuploidy in human glioblastoma cell lines [55]. Lawrence et al. recently identified *STAG2* as one of the 12 genes that were mutated at a substantially high frequency in at least four cancer types through examining the exome sequencing data of 4,742 human cancer samples across 21 cancer types [30]. Taken together, we provided statistical evidence in that hypermutation of the cancer driver genes on the inactive X chromosome may be a general feature in the female cancer genomes [52]. Further investigation on this feature is warranted.

## Discussion

Several previous studies showed several lines of strong biological evidences in that a single gene may shape subsequent evolution of the human cancer genome [8,11,13]. Such evidence motivated us to develop a mathematical model that can quantitatively measure a gene-gene pair to be co-mutated and highly co-expressed simultaneously in a given cancer type. Here, we proposed the gene gravity model based on Newton’s law of gravitation to study the cancer genome evolution by the systematic integration of ~3,000 cancer genome transcription and somatic mutation profiles from TCGA under molecular network architecture knowledge. It is worth noting that some factors, such as gene length, network topology (e.g. connectivity), high mutation rate on the cancer driver genes, and high PCC value for the particular genes, may affect the performance of the gene gravity model.

Longer genes would be more likely to harbor mutations, increasing the false positive rate during cancer genomic analysis [28,32]. We investigated the correlation of the gene ave*G* score with gene cDNA length collected from Tamborero et al. [56]. We removed two longest human genes (*TTN* and *MUC16*) because no evidence has been found in cancer yet [28,32]. We observed a moderate correlation between gene ave*G* score and cDNA length in the 9 cancer types (S18 Fig). For BRCA, the correlation is 0.21 between gene ave*G* score and gene cDNA length (p < 2.2 × 10^−16^). In addition, we recalculated the ave*G* score by using the average mutation density (M/L, here M is the number of mutations for a given gene in a specific cancer type) per base pair in each cancer type normalized by gene cDNA length (L). We could reproduce the results (S19 Fig), since the new results are nearly the same to those presented in S2–S10 Figs.

We next examined whether the gene connectivity and gene average co-expression correlation, such as “party hub” in the network [57], contribute to the performance of the gene gravity model. We found that gene ave*G* score significantly correlates with gene connectivity in all of the 9 cancer types (S20 Fig). For BRCA, the correlation is 0.40 between the gene ave*G* score and gene connectivity in PIN (p < 2.2 × 10^−16^, F-statistics, S20 Fig). Thus, a gene with high connectivity may create a higher cancer genome evolution rate. Additionally, we investigated the relationship between the gene ave*G* and the average gene co-expression coefficient (avePCC). We calculated a gene avePCC using (*ρ*)_*i*_ = ∑_*j*_ *PCC*_*ij*_ / *n* between gene *i* and gene *j* (*j* belongs to the set of gene *i*’s interacting partners (n) in PIN) based on the absolute value of PCC for each gene-gene pair. We found a moderately positive correlation between gene ave*G* score and its avePCC across 9 cancer types (p < 2.2 × 10^−16^, S21 Fig). Finally, we further examined whether we could reproduce the results using 4 features: high connectivity, high avePCC, gene length, and high mutation rate. For comparison, we separated genes into 3 categories based on the range of the ave*G* score. As shown in S22 Fig, for each of these 4 features, the distribution of ave*G* score cannot simply separate 3 different ave*G* categories: low, middle, and high groups. In a previous study, we found a positive correlation of protein connectivity with the number of nonsynonymous somatic mutations across 12 cancer types [23]. Thus, the current observation is consistent with our previous study that network-attacking perturbations due to somatic mutations occurring in the network hubs of the cancer interactome play important roles during tumor emergence and evolution [23].

There are some ultra-mutated tumor samples in various cancer types, such as UCEC or COAD. For example, a small number of tumor samples can contribute to a large proportion (e.g., 40%) of total somatic mutations observed in the whole cancer cohort [29]. We removed 18 ultra-mutated tumor samples in UCEC and 31 ultra-mutated tumor samples in COAD based on a previous study [29]. We then used the remaining tumor samples to perform the same analyses. As shown in S23 and S24 Figs, we could reproduce the results, since the new results are nearly the same to those presented in Fig 2 and S2–S10 Figs. Thus, ultra-mutated tumor samples only had a minor influence on the performance of gene gravity model.

There are several limitations in the current model. First, for the TCGA data, its inherent static nature gives only a single time point analysis, and we are unable to map specific genome or protein changes to the individual cells or cell populations through whole-tumor tissue analysis. Second, tumor heterogeneity and environmental factors may increase the data bias. For example, we did not find a substantial pattern indicating that the attractive gravitation for oncogenes is very stronger than that of non-oncogenes in GBM, HNSC, LUAD, or LUSC (S12 Fig). One possible explanation is that environmental factors (e.g., smoking) may accelerate cancer genome evolution. We separated TCGA patients into smokers and never-smokers in LUAD and LUSC, and performed the same analysis by quantifying the gene gravity model. As expected, we found that the attractive gravitation of oncogenes is significantly stronger than that of non-oncogenes for never-smokers in LUAD or LUSC (S25 Fig). However, the attractive gravitation of oncogenes is marginally higher than that of non-oncogenes for smokers in LUAD or LUSC (S25 Fig). Third, we used a broad context molecular network to derive the gene gravity model. However, current molecular network architectures do not completely represent the natural genetic profiles of cells. In the future, we may improve the gene gravity model in the following ways: (i) integrate single-cell data, including single-cell gene expression and next-generation sequencing data, to explore the dynamic features of cells and reduce the influence of tumor purity and tumor heterogeneity [58–61]; (ii) address cancer genetic network signatures by using large-scale genetic interaction profiles [62]; and, (iii) integrate panomics data resources, including the chromatin interaction network, copy number variation, proteomics, and DNA methylation profiles, to explore genomic instability more deeply and identify putative cancer driver genes [32,63]. Finally, we plan to use an insulated heat diffusion process implemented in a previous study [64] to consider the significance of the cancer driver genes regardless of network topology (e.g. connectivity). In summary, this study reaffirms the power and value of TCGA panomic data in investigating fundamental cancer biology questions, such as somatic mutation-driven cancer genome evolution.

## Materials and Methods

### Construction of molecular network

We downloaded the PPI data and constructed a large-context PIN from two sources: InnateDB [65] and the Protein Interaction Network Analysis (PINA) platform [66]. InnateDB contained more than 196,000 experimentally validated molecular interactions in human, mouse, and bovine models. PINA (v2.0) is a comprehensive PPI database that integrates six high-quality public databases. We implemented three data cleaning steps. First, we defined an interaction as being high-quality if it was experimentally validated in human models through a well-defined experimental protocol. The interactions that did not satisfy this criterion were discarded. Second, we annotated all protein-coding genes using gene Entrez ID, chromosome location, and the gene official symbols from the NCBI database (http://www.ncbi.nlm.nih.gov/). Finally, duplicated or self-loop interactions were removed. In total, we obtained 113,473 unique interactions connecting 13,579 protein-coding genes (S2 Table).

### Collection of RNA-Seq data and gene co-expression analysis

We collected RNA-Seq data (V2) from 3,487 tumor samples across 9 cancer types from TCGA (http://cancergenome.nih.gov/). These 9 cancer types consisted of BRCA, COAD, GBM, HNSC, KIRC, LUAD, LUSC, OV, and UCEC (S1 Table). In this study, we implemented two criteria to select the genes that were expressed: (i) in a sample, we filtered out the genes whose mRNA expression was below the 20% of all mRNAs ordered by their expression level; and (ii) we further filtered out the genes that expressed in less than 20% of samples in whole expression matrix. We also extracted RNA-Seq V2 data for smokers and never-smokers in LUAD and LUSC, and for the male and female genomes in GBM from TCGA (January 05, 2015) using the R package implemented in TCGA-Assembler [67]. Finally, we calculated the Pearson Correlation Coefficient (PCC) for each gene-gene pair and mapped the PCC value of each gene-gene pair onto above PIN to construct 9 CePINs for the 9 cancer types (Fig 1A).

### Somatic mutations in 3,000 cancer genomes

We collected somatic mutation profiles for 2,946 cancer exomes in 9 cancer types (S1 Table). In total, we obtained 277,370 nonsynonymous somatic mutations on the protein-coding regions in ~18,000 genes. The details of preprocessing of mutation data are provided in Kandoth et al. [29]. We also extracted somatic missense mutations for smokers and never-smokers in LUAD and LUSC, and for the male and female genomes in GBM from TCGA (January 05, 2015) using the R package implemented in TCGA-Assembler [67].

### Gene gravity model

#### Mutation propagation

We mapped the somatic mutations in each cancer type onto PIN (Fig 1B). We used a network smoothing method [68] to spread the mutations across the whole network for each cancer type. In this framework, we applied the random walk with restart algorithm to calculate the cumulative mutations for each gene (Fig 1C). We denoted $\vec{{\text{M}}_{\text{(t)}}}$ as the mutation vector at iteration step *t*, and the propagation process is described as $\vec{{\text{M}}_{\text{(t}+\text{1)}}}=\alpha {\text{P}}^{\text{T}}\vec{{\text{M}}_{\text{(t)}}}+(1-\alpha )\vec{{\text{M}}_{\text{0}}}$, where $\vec{{\text{M}}_{\text{0}}}$ is a n×1 vector (*n* is the number of genes in the network) with the *i*-th element equal to the cumulative mutation number of the gene through all samples for each cancer type. P^T^ is the transition matrix with P_ij_ = 1/k_i_ if *i* and *j* are connected, otherwise P_ij_ = 0 (k_i_ is the connectivity of gene *i* in the network); and *α* is a tuning parameter driving the restart probability of the random walk process. The mutations transmit to a random neighbor with the probability *α* and returns to the initial gene with the probability (1−*α*). The theoretical solution is straightforward when *α* ∈ (0, 1), as $\vec{{\text{M}}_{\text{(}\infty \text{)}}}=(1-\alpha ){\left(1-\alpha {\text{P}}^{\text{T}}\right)}^{-1}\vec{{\text{M}}_{\text{(0)}}}$ [69]. We used the iteration of the propagation function until $\vec{{\text{M}}_{\text{t}}}$ converged by the convergence condition ${\left\|\vec{{\text{M}}_{\text{(t}+\text{1)}}}-\vec{{\text{M}}_{\text{(t)}}}\right\|}^{2}<10^{-6}$ for a large network calculation.

For *α* = 1, the stationary solution of $\vec{{\text{M}}_{\text{t}}}$ is k_i_/2*N*_*L*_ (*N*_*L*_ is the total edges in PIN), which is determined only by the network structure. When *α* = 0, $\vec{{\text{M}}_{\text{t}}}$converges to $\vec{{\text{M}}_{\text{0}}}$ and only depends on the cumulative mutations through the samples. Here α is an important parameter in mutation propagation. There is no propagation when α = 0; while the M value of a gene is purely determined by the network structure when α = 1. We examined the influence of different α value (0.1 to 0.9) in BRCA. As shown in S26 Fig, the α value (after α > 0.7) affects the results slightly. Following the propagation process by setting α = 0.7, we built 9 mutation PINs for the 9 cancer types respectively by incorporating nonsynonymous somatic mutations into each PIN to yield a cumulative mutations for each gene. In addition, we also performed mutation propagation by setting α = 0.2. We could reproduce the results (S27 Fig) by setting α = 0.2 when compared to that by setting α = 0.7 (Fig 2).

#### Gene gravity model

The gravity model derived from Newton’s law of gravitation has been used in several fields, e.g., population migration [70]. In a classical gravity model, the gravitation of two bodies is proportional to the product of their masses and inversely proportional to the square of the distance between them, that is, $\text{G}=\text{k}\frac{{\text{m}}_{\text{1}}{\text{m}}_{\text{2}}}{{\text{r}}^{\text{2}}}$, where m_1_ and m_2_ represent the masses of two bodies, *r* represents the distance between them, and *k* is the gravitation constant. Here, we proposed a model to derive the genetic interaction between two genes in the given cancer type. We assumed that the genetic interaction between genes *i* and *j* follows a gravity model. Our model is ${\text{G}}_{\text{ij}}=\text{k}\frac{{\text{M}}_{\text{i}}{\text{M}}_{\text{j}}}{{\text{r}}_{\text{ij}}^{\text{2}}}$, where M_i_ represents the cumulative mutations of gene *i* according to the mutation propagation method, *r*_*ij*_ represents the “biological distance” between genes *i* and *j*, and *k* was assumed to be 1. For each cancer type, we used the PCC values of gene co-expression pairs from RNA-Seq data to evaluate the gene-gene “biological distance” ${\text{r}}_{\text{ij}}=\frac{1}{{\text{PCC}}_{\text{ij}}}$. From the definition of *r*_*ij*_, a high PCC indicates a short distance, and vice versa. Following the definition of *G*_ij_, two genes having large cumulative mutations and high gene co-expression would exhibit a stronger genetic interaction (high *G* score) with each other.

### Categories of gene sets

#### Cancer driver genes

We collected a high-quality mutated cancer driver gene set from four large-scale, cancer genome analysis projects [3,28–30], as briefly described below. (i) Lawrence et al. identified 224 significantly mutated genes from 4,742 human cancer exomes in 21 cancer types using the MutSig method [30]. (ii) Vogelstein et al. identified 125 mutated cancer genes from the genome-wide sequencing studies of 3,284 tumors using the 20/20 rule [3]. (iii) Kandoth et al. identified 127 significantly mutated genes from 3,281 tumors across 12 cancer types [29]. (iv) Tamborero et al. identified 291 high-confidence mutated cancer driver genes in 3,205 tumors from 12 different cancer types using MutSig, OncodriveFM, OncodriveCLUST, and ActiveDriver methods [28]. We utilized a union of four driver gene sets, resulting in a total of 614 cancer driver genes (S3 Table).

#### DNA repair genes

We collected 153 DNA repair genes from the REPAIRtoire database [26]. DNA repair enzymes continuously monitor chromosomes to correct damaged nucleotide residues generated by exposure to carcinogens and cytotoxic agents [25], whose processes are crucial for the maintenance of genetic information in the cancer genome.

#### Chromatin regulation factors

We compiled 176 CRFs from a previous study [27]. CRFs regulate chromatin structure using three distinct processes: the post-translational modification of histone tails, the replacement of core histones by histone variants, and direct structural remodeling by ATP-dependent chromatin-remodeling enzymes. The CRFs that modulate the epigenetic landscape have emerged as potential gatekeepers and signaling coordinators for the maintenance of genome integrity [24].

#### Essential genes

We compiled 2,719 essential genes from the OGEE database [35]. Essential genes, whose knockouts result in lethality or infertility, are important for studying the robustness of a biological system [35].

#### Other cancer genes

First, 487 CGC genes were downloaded from Cancer Gene Census [71] (July 10, 2013). We then annotated oncogenes and TSGs using information from two publicly available databases: CancerGenes [72] and TSGene [73]. In total, we obtained 477 oncogenes and 1,040 TSGs (S3 Table).

### Statistical analysis

All statistical tests were conducted using the R package (v3.0.1, http://www.r-project.org/). The *q* values less than 0.1 were considered statistically significant.

## Supporting Information

S1 Fig The distribution of mutational density on the protein-protein interaction pairs in comparison to the unfiltered interactions relative to the same number of random pairs across 9 cancer types. (PDF)

S2 Fig The complementary cumulative distribution of the gene-gene gravitation score for five different gene sets in breast invasive carcinoma (BRCA). (PDF)

S3 Fig The complementary cumulative distribution of the gene-gene gravitation score for five different gene sets in colon adenocarcinoma (COAD). (PDF)

S4 Fig The complementary cumulative distribution of the gene-gene gravitation score for five different gene sets in glioblastoma multiforme (GBM). (PDF)

S5 Fig The complementary cumulative distribution of the gene-gene gravitation score for five different gene sets in head and neck squamous cell carcinoma (HNSC). (PDF)

S6 Fig The complementary cumulative distribution of the gene-gene gravitation score for five different gene sets in kidney renal clear cell carcinoma (KIRC). (PDF)

S7 Fig The complementary cumulative distribution of the gene-gene gravitation score for five different gene sets in lung adenocarcinoma (LUAD). (PDF)

S8 Fig The complementary cumulative distribution of the gene-gene gravitation score for five different gene sets in lung squamous cell carcinoma (LUSC). (PDF)

S9 Fig The complementary cumulative distribution of the gene-gene gravitation score for five different gene sets in ovarian serous cystadenocarcinoma (OV). (PDF)

S10 Fig The complementary cumulative distribution of the gene-gene gravitation score for five different gene sets in uterine corpus endometrial carcinoma (UCEC). (PDF)

S11 Fig The complementary cumulative distribution (C) of the gene-gene gravitation score (*G*) for Cancer Gene Census (CGC) genes in lung adenocarcinoma (LUAD) and lung squamous cell carcinoma (LUSC) smoker versus never-smoker (Nonsmoker) patients. (PDF)

S12 Fig The complementary cumulative distribution (C) of the attractive gene-gene gravitation (*G*) scores for oncogenes across 9 cancer types. (PDF)

S13 Fig The complementary cumulative distribution (C) of the repulsive gene-gene gravitation (*G*) scores for oncogenes across 9 cancer types. (PDF)

S14 Fig The complementary cumulative distribution (C) of the repulsive gene-gene gravitation (*G*) scores for tumor suppressor genes across 9 cancer types. (PDF)

S15 Fig The complementary cumulative distribution (C) of the attractive gene-gene gravitation (*G*) scores for tumor suppressor genes across 9 cancer types. (PDF)

S16 Fig Venn diagrams showing the relationship between Cancer Gene Census (CGC) genes with (A) chromatin regulation factors (CRFs) and (B) DNA repair genes. (PDF)

S17 Fig The distribution of average gravitation score across 23 chromosomes for 6 cancer types. (PDF)

S18 Fig Correlation between gene average gravitation score and gene length (cDNA length, bp) across 9 cancer types. The correlation r was calculated using Pearson Correlation Coefficient, and the p-value was calculated using F-statistics.
(PDF)

S19 Fig Box plot shows new gene-gene pair gravitation (*G*) score distribution when using the average mutation rate (M/L, here M is the mutation frequency for a given genes in a specific cancer type) per base pair (bp) in each cancer type normalized by gene cDNA length (L) for five gene sets across 9 cancer types. (PDF)

S20 Fig Correlation between gene average gravitation score and gene connectivity in protein interaction network across 9 cancer types. The correlation r was calculated using Pearson Correlation Coefficient, and the p-value was calculated using F-statistics.
(PDF)

S21 Fig Correlation between gene average gravitation score and average co-expression coefficient (avePCC) across 9 cancer types. The correlation r was calculated using Pearson Correlation Coefficient, and the p-value was calculated using F-statistics.
(PDF)

S22 Fig The relationship between gene average gravitation (ave*G*) scores with four features: average Pearson Correlation Coefficient (avePCC), mutation rate, gene cDNA length, and gene connectivity (degree) in 9 cancer types. (PDF)

S23 Fig The performance of the gene gravity model after removing 31 ultramutated tumor samples in colon adenocarcinoma (COAD). (PDF)

S24 Fig The performance of the gene gravity model after removing 18 ultramutated tumor samples in uterine corpus endometrial carcinoma (UCEC). (PDF)

S25 Fig The complementary cumulative distribution (C) of the attractive gene-gene gravitation score (*G*) for oncogenes versus non-oncogenes in lung adenocarcinoma (LUAD) and lung squamous cell carcinoma (LUSC) smoker and never-smoker (Nonsmoker) patients. (PDF)

S26 Fig The influence of gene average gravitation (ave*G*) scores accompanying by an important parameter Alpha during mutation network propagation in breast invasive carcinoma (BRCA). (PDF)

S27 Fig Box plots of gene average gravitation (ave*G*) score for five gene sets across 9 cancer types by setting alpha = 0.2 during mutation network propagation. (PDF)

S1 Table The statistics of transcription (RNA-seq) and somatic mutation profiles across 9 cancer types used in this study. (PDF)

S2 Table The protein interaction network used in this study. (ZIP)

S3 Table Lists of five gene sets: 153 DNA repair genes, 176 chromatin regulation factors, 614 cancer driver genes, 487 Cancer Gene Census (CGC) genes, 477 oncogenes, and 1,040 tumor suppressor genes. (ZIP)

S4 Table The gene average gravitation score for 5 gene sets across 9 cancer types. (PDF)

S5 Table The average gravitation score of CRF cancer driver genes, non-CRF cancer driver genes and non-driver CRF genes. (PDF)

S6 Table The average gravitation score of chromatin regulation factor (CRF) and Cancer Gene Census (CGC) genes, non-CRF CGC genes and non-CGC CRF genes. (PDF)

S7 Table The average gravitation score of DNA repair cancer driver genes, non-DNA repair cancer driver genes, and non-driver DNA repair genes. (PDF)

S8 Table The average gravitation score of DNA repair Cancer Gene Census (CGC) genes, non-DNA repair CGC genes, and non-CGC DNA repair genes. (PDF)

S9 Table The enrichment analysis of the top 100 genes that have the highest gene average gravitation score between cancer driver genes and non-driver genes. (PDF)

S10 Table The enrichment analysis of the top 100 genes that have the highest gene average gravitation score between Cancer Gene Census (CGC) and non-CGC genes. (PDF)

S11 Table The enrichment analysis of the top 100 genes that have the highest gene average gravitation score between chromatin regulation factors (CRFs) and non-CRFs. (PDF)

S12 Table The enrichment analysis of the top 100 genes that have the highest gene average gravitation score between DNA repair genes and non-DNA repair genes. (PDF)

S13 Table The average gene gravitation scores across 9 cancer types. (ZIP)
