# Supplementary material for: A Gene Gravity Model for the Evolution of Cancer Genomes: A Study of 3,000 Cancer Genomes across 9 Cancer Types
Source: PLoS Comput Biol. 2015 Sep 9;11(9):e1004497. doi: 10.1371/journal.pcbi.1004497 (PMC4564226; doi:10.1371/journal.pcbi.1004497)
Supplement: S1 Fig — (PDF) [file pcbi.1004497.s001.pdf]

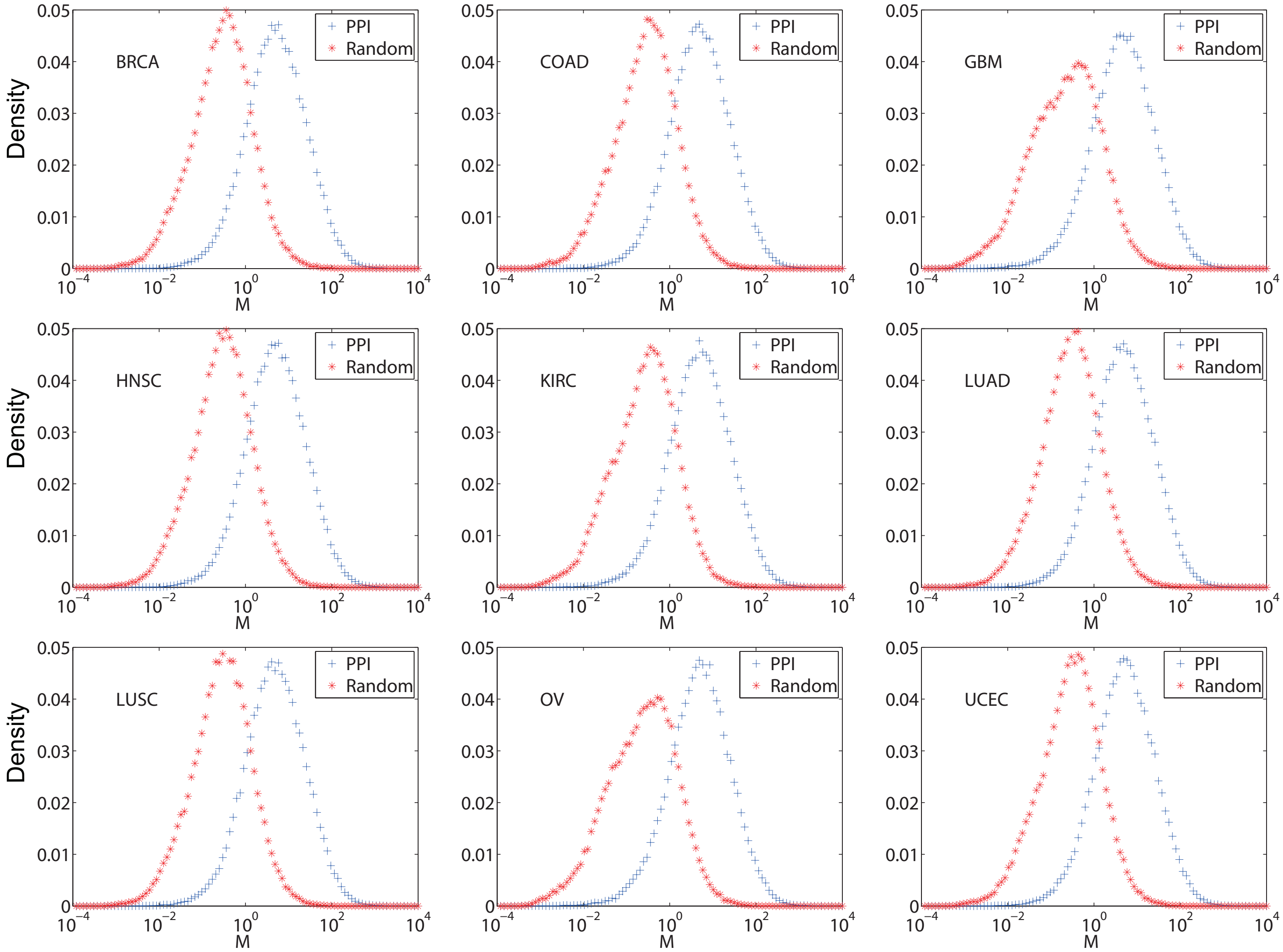

Fig. S1. The distribution of mutational signal (M=M1×M2, see the section of Methods and Materials) values of the protein-protein interaction (PPI) pairs in the protein interaction network in comparison to the unfiltered interactions relative to the same number of random pairs across 9 cancer types. We found that PIN is significantly more enriched for high mutation rates than random pairs across 9 cancer types ($q < 2.2\times10^{-16}$, Wilcoxon sum-rank test adjusted by R-package multiple test. BRCA: breast invasive carcinoma, COAD: colon adenocarcinoma, GBM: glioblastoma multiforme, HNSC: head and neck squamous cell carcinoma, KIRC: kidney renal clear cell carcinoma, LUAD: lung adenocarcinoma, LUSC: lung squamous cell carcinoma, OV: ovarian serous cystadenocarcinoma, and UCEC: uterine corpus endometrial carcinoma.
