# Supplementary material for: A Gene Gravity Model for the Evolution of Cancer Genomes: A Study of 3,000 Cancer Genomes across 9 Cancer Types
Source: PLoS Comput Biol. 2015 Sep 9;11(9):e1004497. doi: 10.1371/journal.pcbi.1004497 (PMC4564226; doi:10.1371/journal.pcbi.1004497)
Supplement: S9 Fig — (PDF) [file pcbi.1004497.s009.pdf]

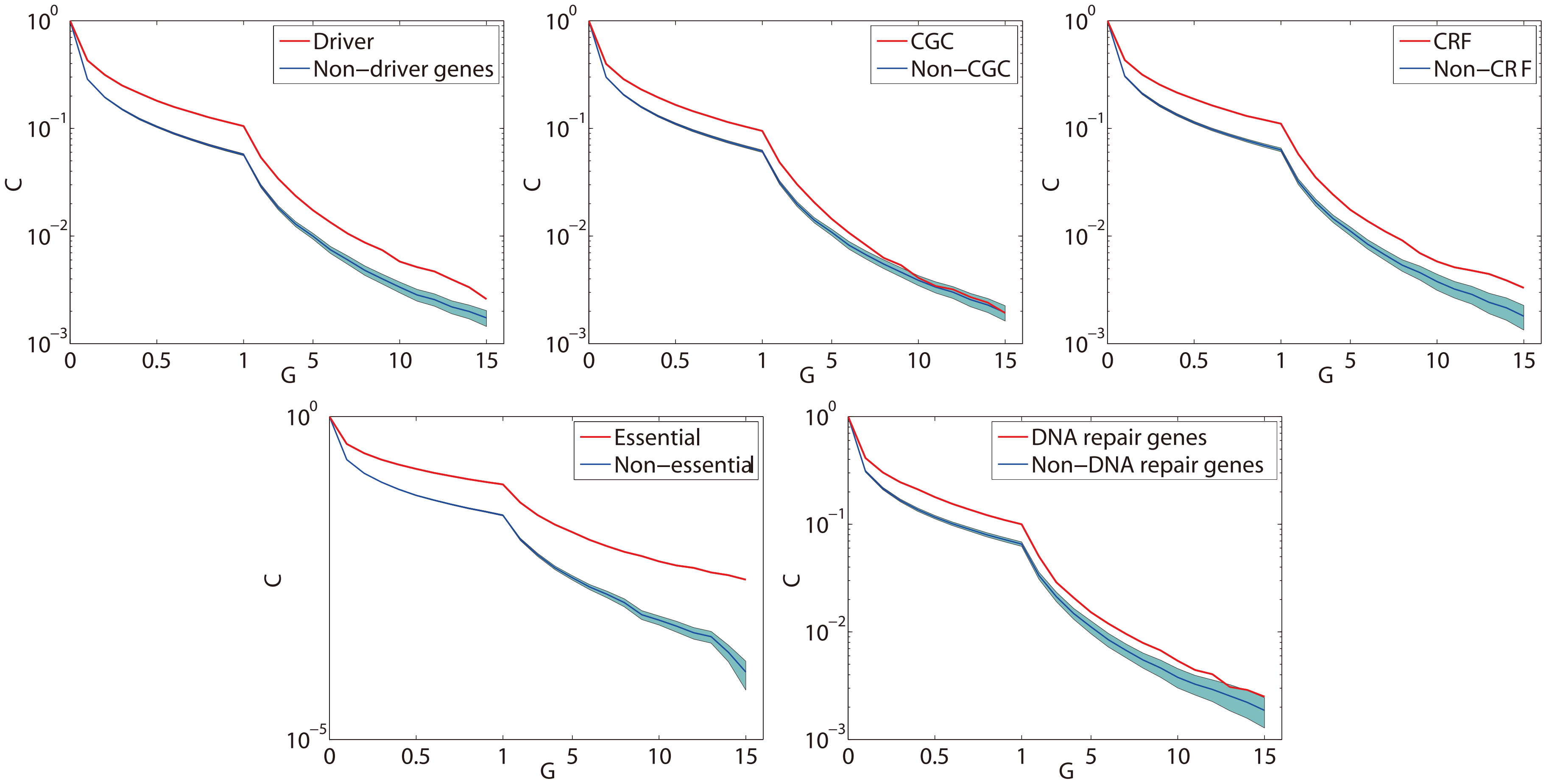

Fig. S9. The complementary cumulative distribution (C) of the gene-gene gravitation (G) score for five different gene sets: cancer driver gene (Driver), Cancer Gene Census (CGC), chromatin regulation factors (CRF), essential genes, and DNA repair genes in ovarian serous cystadenocarcinoma (OV). The number of gene-gene pairs for blue line is equal to red line by random sampling 100 times. The shadow of blue line represent the standard deviation.
