# Supplementary material for: A Gene Gravity Model for the Evolution of Cancer Genomes: A Study of 3,000 Cancer Genomes across 9 Cancer Types
Source: PLoS Comput Biol. 2015 Sep 9;11(9):e1004497. doi: 10.1371/journal.pcbi.1004497 (PMC4564226; doi:10.1371/journal.pcbi.1004497)
Supplement: S11 Fig — (PDF) [file pcbi.1004497.s011.pdf]

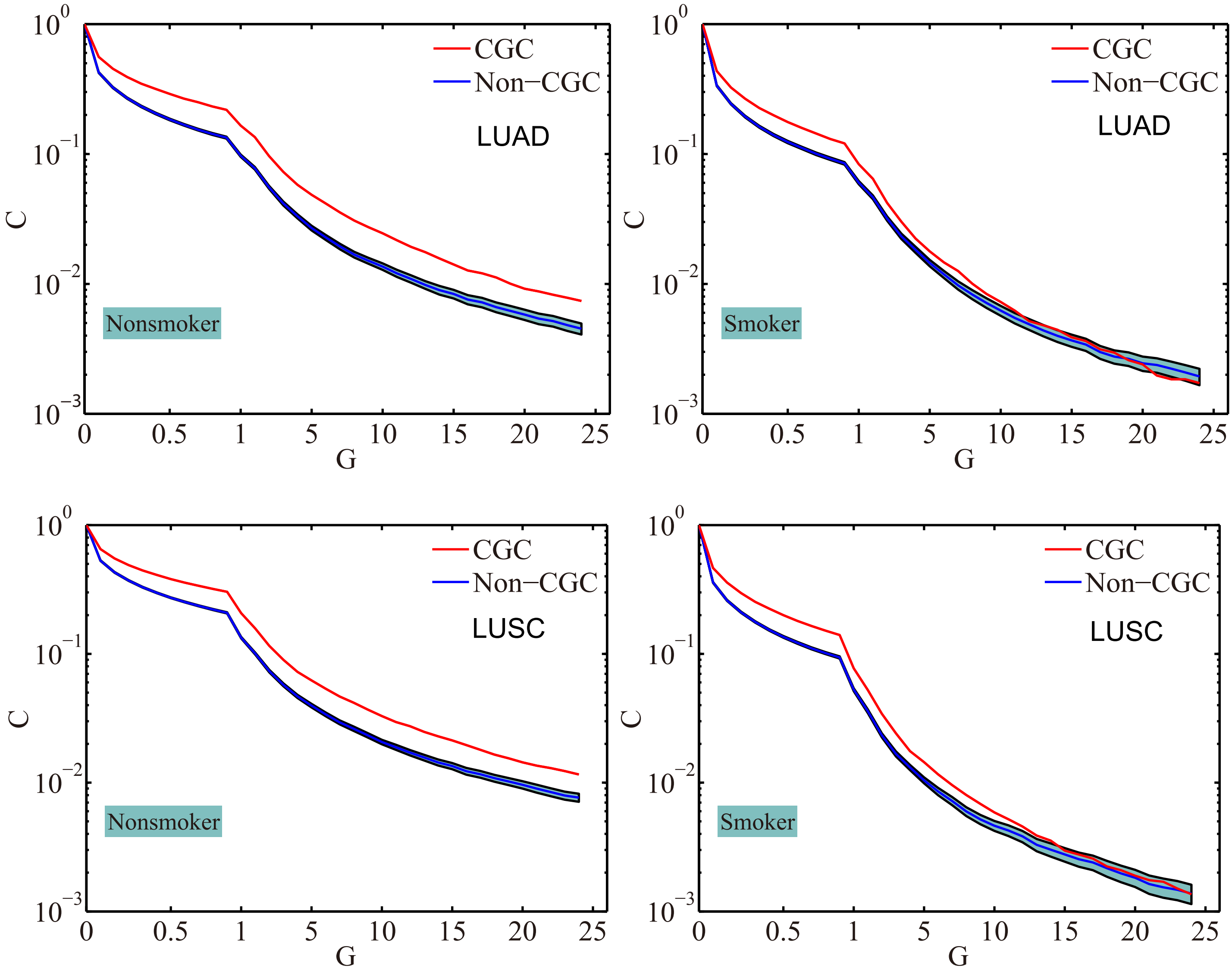

Fig. S11. The complementary cumulative distribution (C) of the gene-gene gravitation score (G) for Cancer Gene Census (CGC) genes in lung adenocarcinoma (LUAD) and lung squamous cell carcinoma (LUSC) smoker and never-smoker (Nonsmoker) patients. The number of gene-gene pairs for blue line is equal to red line by random sampling 100 times. The shadow of blue line represent the standard deviation.
