# Supplementary material for: A Gene Gravity Model for the Evolution of Cancer Genomes: A Study of 3,000 Cancer Genomes across 9 Cancer Types
Source: PLoS Comput Biol. 2015 Sep 9;11(9):e1004497. doi: 10.1371/journal.pcbi.1004497 (PMC4564226; doi:10.1371/journal.pcbi.1004497)
Supplement: S14 Fig — (PDF) [file pcbi.1004497.s014.pdf]

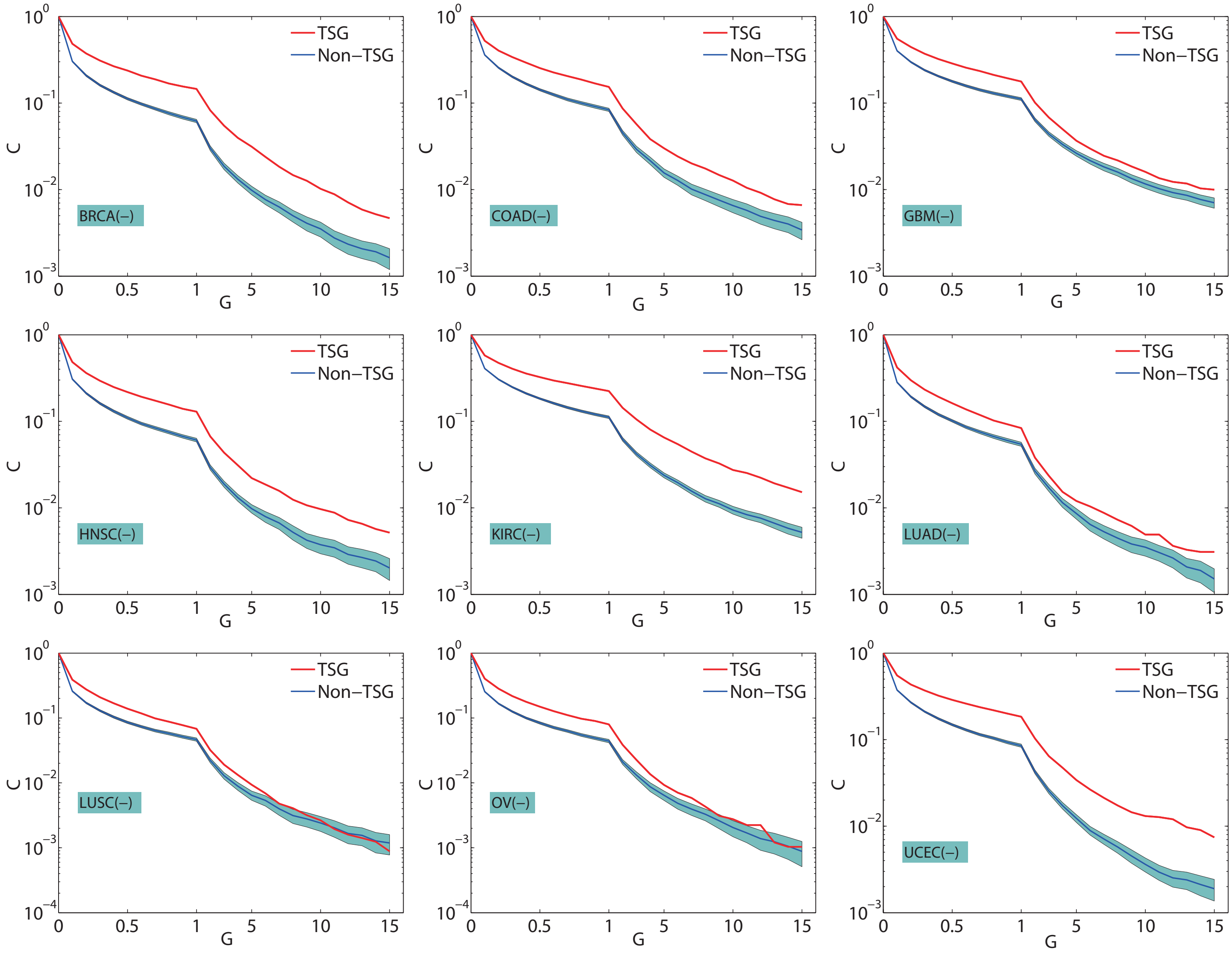

Fig. S14. The complementary cumulative distribution (C) of the repulsive gene-gene gravitation score (G) for tumor suppressor genes (TSG) across 9 major cancer types: breast invasive carcinoma (BRCA), colon adenocarcinoma (COAD), glioblastoma multiforme (GBM), head and neck squamous cell carcinoma (HNSC), kidney renal clear cell carcinoma (KIRC), lung adenocarcinoma (LUAD), lung squamous cell carcinoma (LUSC), ovarian serous cystadenocarcinoma (OV), and uterine corpus endometrial carcinoma (UCEC). The number of repulsive gene-gene pairs for blue line is equal to red line by random sampling 100 times. The shadow of blue line represent the standard deviation.
