# Supplementary material for: A Gene Gravity Model for the Evolution of Cancer Genomes: A Study of 3,000 Cancer Genomes across 9 Cancer Types
Source: PLoS Comput Biol. 2015 Sep 9;11(9):e1004497. doi: 10.1371/journal.pcbi.1004497 (PMC4564226; doi:10.1371/journal.pcbi.1004497)
Supplement: S16 Fig — (PDF) [file pcbi.1004497.s016.pdf]

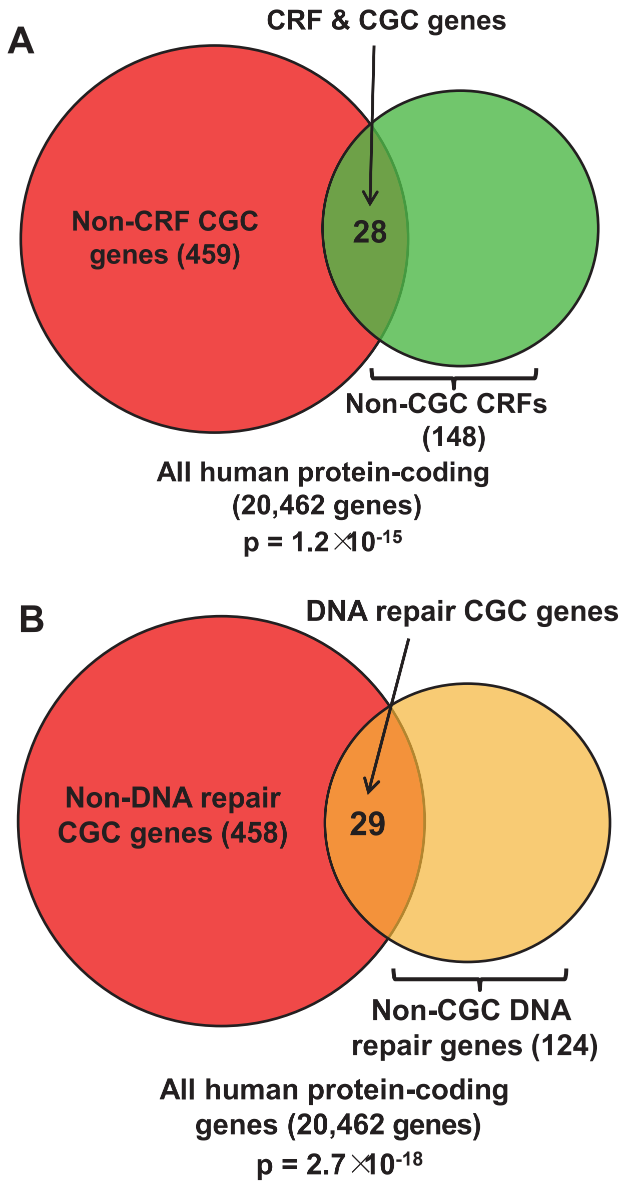

Fig. S16. The left Venn diagrams show the enrichment of Cancer Gene Census (CGC) genes in (A) chromatin regulation factors (CRFs) and (B) DNA repair genes by comparing to the whole human genome (20,462 protein-coding genes collecting from National Center for Biotechnology Information database. The p-value (p) was calculated using Fisher's exact test.
