# Supplementary material for: A Gene Gravity Model for the Evolution of Cancer Genomes: A Study of 3,000 Cancer Genomes across 9 Cancer Types
Source: PLoS Comput Biol. 2015 Sep 9;11(9):e1004497. doi: 10.1371/journal.pcbi.1004497 (PMC4564226; doi:10.1371/journal.pcbi.1004497)

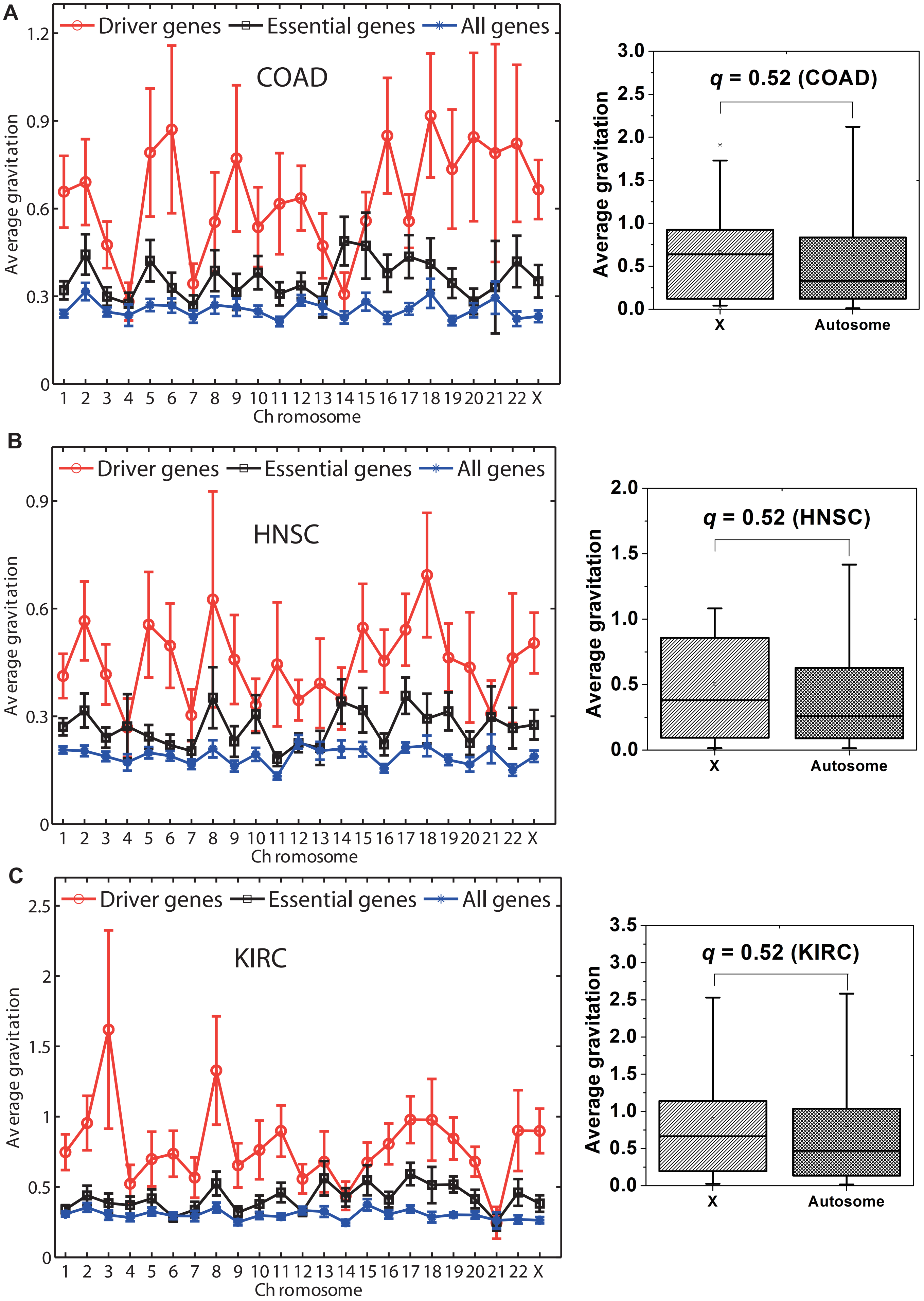

Fig. S17. The average gravitation score distribution of cancer driver genes, essential genes, and all genes across chromosomes for 6 cancer types: (A) COAD: colon adenocarcinoma, (B) HNSC: head and neck squamous cell carcinoma, (C) KIRC: kidney renal clear cell carcinoma, (D) LUAD: lung adenocarcinoma, (E) LUSC: lung squamous cell carcinoma, and (F) OV: ovarian serous cystadenocarcinoma. Left box plot shows the cancer driver gene average gravitation between X chromosome and the entire 22 autosomes. The p-values are calculated by the Wilcoxon sum-rank test and corrected by R package multiple test.

Fig. S17. continue...

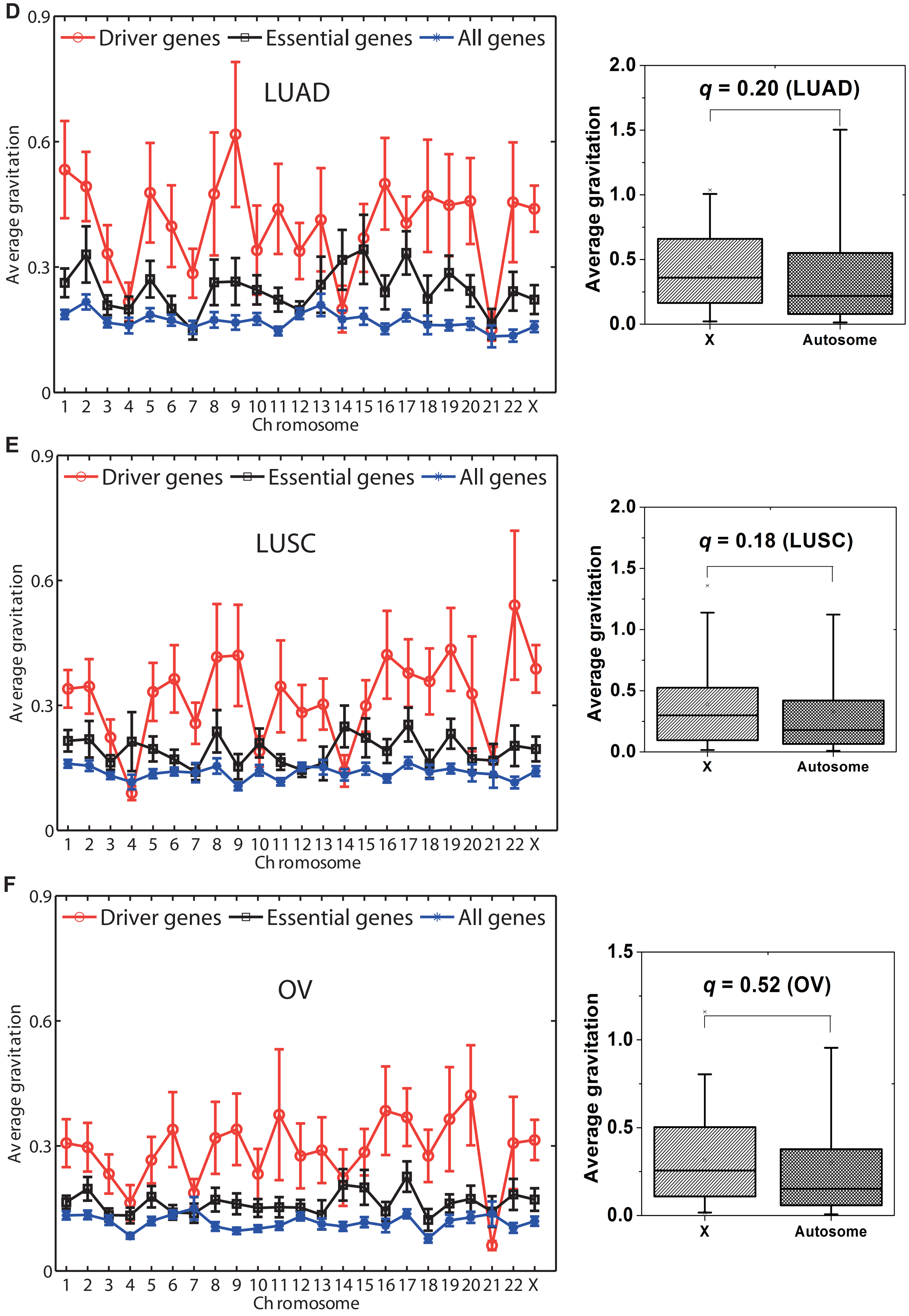

Supplement: S17 Fig — (PDF) [file pcbi.1004497.s017.pdf]
