# Supplementary material for: A Gene Gravity Model for the Evolution of Cancer Genomes: A Study of 3,000 Cancer Genomes across 9 Cancer Types
Source: PLoS Comput Biol. 2015 Sep 9;11(9):e1004497. doi: 10.1371/journal.pcbi.1004497 (PMC4564226; doi:10.1371/journal.pcbi.1004497)
Supplement: S19 Fig — (PDF) [file pcbi.1004497.s019.pdf]

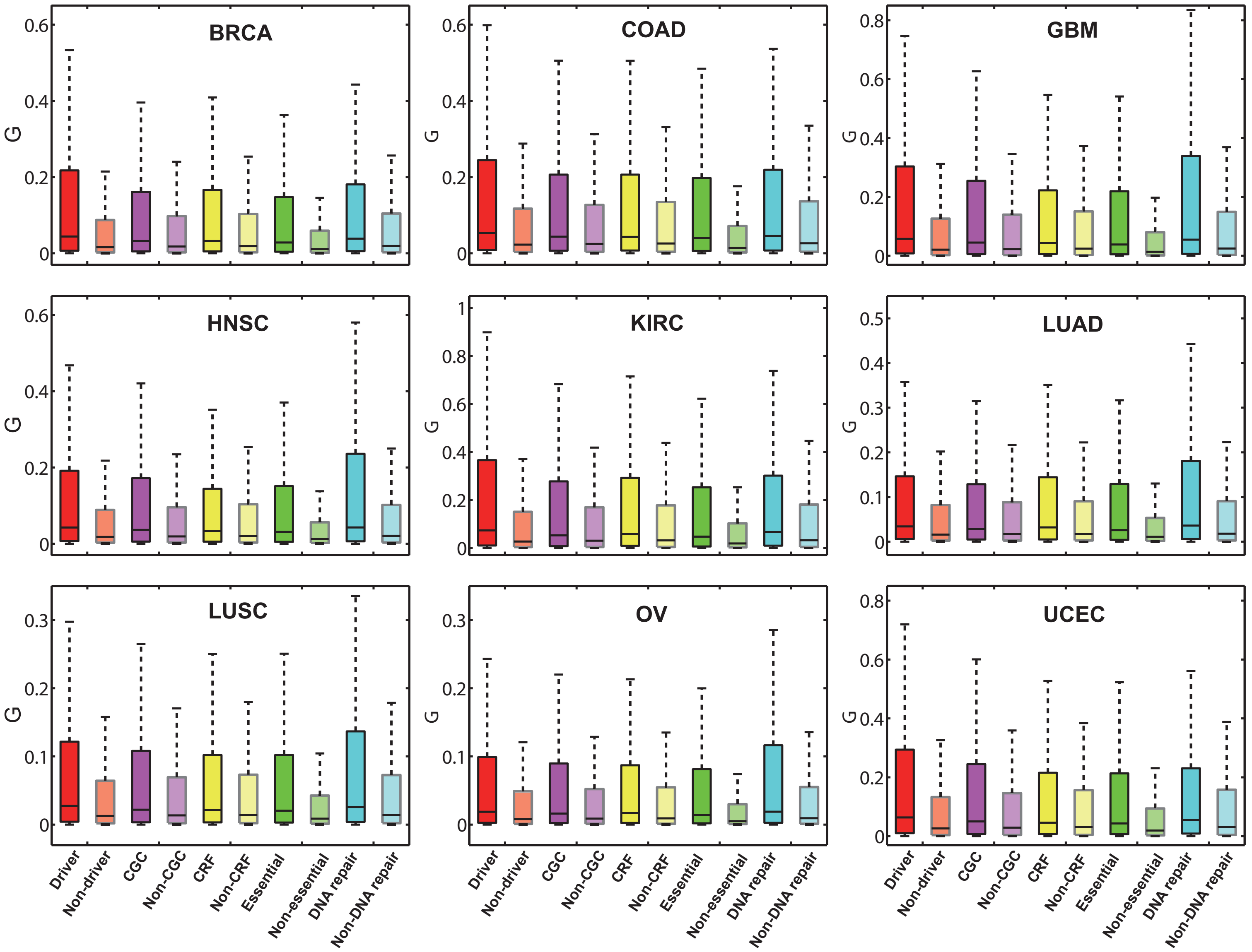

Fig. S19. Box plot of new gene-gene gravitation (G) score when considering the gene cDNA length for five gene sets across 9 cancer types. The new gene-gene G score was recalculated using the average mutation rate per base pair (bp) in each cancer type normalized by gene cDNA length. Red: cancer driver genes (driver) versus non-cancer driver genes (non-driver); purple: Cancer Gene Census genes versus non-CGC genes; yellow: chromatin regulation factors (CRFs) versus non-CRFs; green: essential genes (essential) versus non-essential genes (non-essential); blue: DNA repair genes versus non-DNA repair genes. BRCA: breast invasive carcinoma, COAD: colon adenocarcinoma, GBM: glioblastoma multiforme, HNSC: head and neck squamous cell carcinoma, KIRC: kidney renal clear cell carcinoma, LUAD: lung adenocarcinoma, LUSC: lung squamous cell carcinoma, OV: ovarian serous cystadenocarcinoma, and UCEC: uterine corpus endometrial carcinoma.
