# Supplementary material for: A Gene Gravity Model for the Evolution of Cancer Genomes: A Study of 3,000 Cancer Genomes across 9 Cancer Types
Source: PLoS Comput Biol. 2015 Sep 9;11(9):e1004497. doi: 10.1371/journal.pcbi.1004497 (PMC4564226; doi:10.1371/journal.pcbi.1004497)
Supplement: S20 Fig — The correlation r was calculated using Pearson Correlation Coefficient, and the p-value was calculated using F-statistics. (PDF) [file pcbi.1004497.s020.pdf]

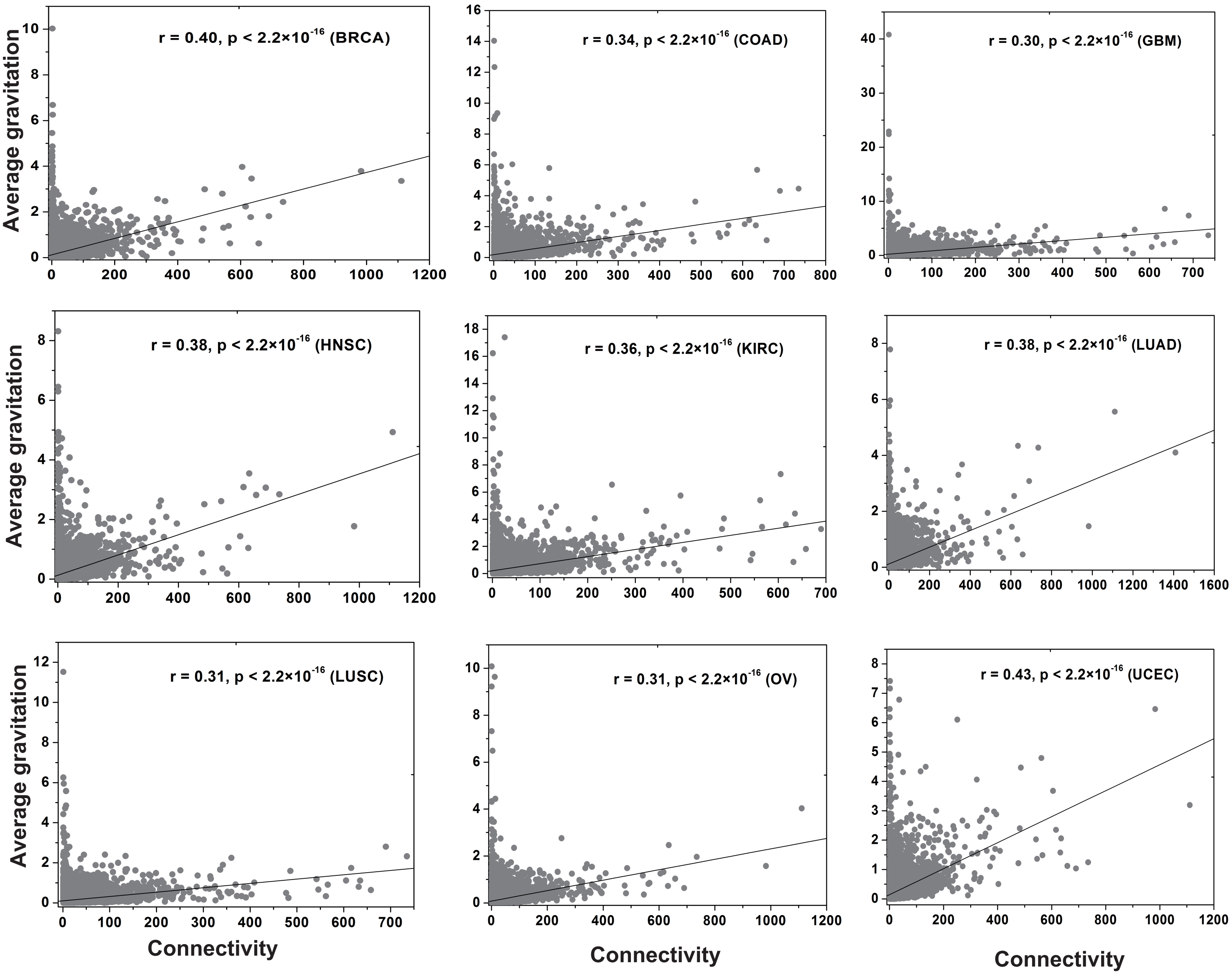


**Fig. S20.** Correlation between gene average gravitation score and gene connectivity in protein interaction network across 9 cancer types. The p-value was calculated using F-statistics. BRCA: breast invasive carcinoma, COAD: colon adenocarcinoma, GBM: glioblastoma multiforme, HNSC: head and neck squamous cell carcinoma, KIRC: kidney renal clear cell carcinoma, LUAD: lung adenocarcinoma, LUSC: lung squamous cell carcinoma, OV: ovarian serous cystadenocarcinoma, and UCEC: uterine corpus endometrial carcinoma.
