# Supplementary material for: A Gene Gravity Model for the Evolution of Cancer Genomes: A Study of 3,000 Cancer Genomes across 9 Cancer Types
Source: PLoS Comput Biol. 2015 Sep 9;11(9):e1004497. doi: 10.1371/journal.pcbi.1004497 (PMC4564226; doi:10.1371/journal.pcbi.1004497)
Supplement: S22 Fig — (PDF) [file pcbi.1004497.s022.pdf]

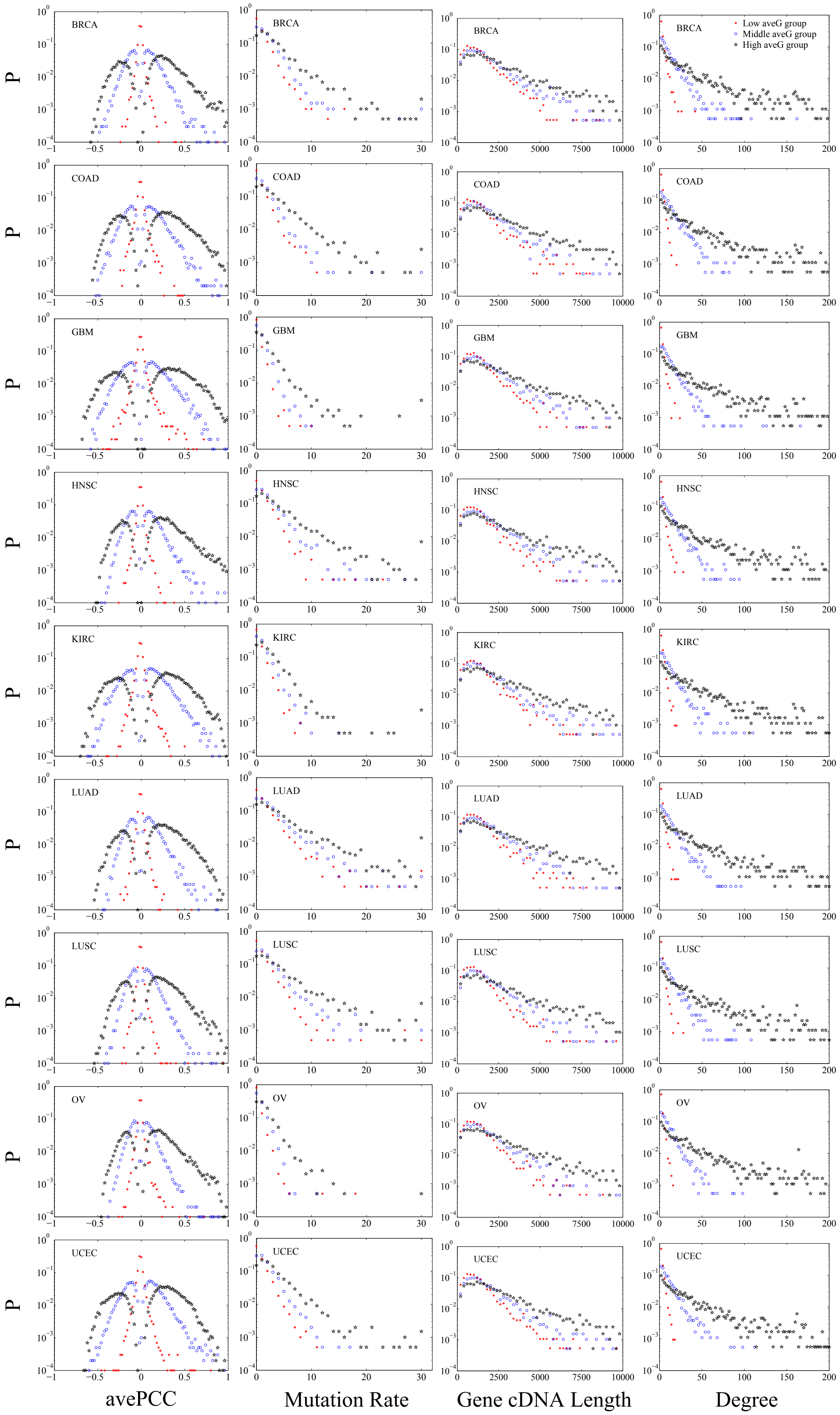

Fig. S22. The relationship between gene average gravitation (aveG) scores with four features: average Pearson Correlation Coefficient (avePCC), mutation rate, gene cDNA length, and gene connectivity (degree) in 9 cancer types. We separated all genes into three different categorgies based on thier aveG scores: low aveG group (down 2,000 genes having the lowest aveG scores), middle aveG group (middle 2000 genes having the the aveG scores ranked bewteen 4,500 to 6,500), high aveG group (top 2,000 genes have the highest aveG scores). P: probability.
