# Supplementary material for: A Gene Gravity Model for the Evolution of Cancer Genomes: A Study of 3,000 Cancer Genomes across 9 Cancer Types
Source: PLoS Comput Biol. 2015 Sep 9;11(9):e1004497. doi: 10.1371/journal.pcbi.1004497 (PMC4564226; doi:10.1371/journal.pcbi.1004497)
Supplement: S23 Fig — (PDF) [file pcbi.1004497.s023.pdf]

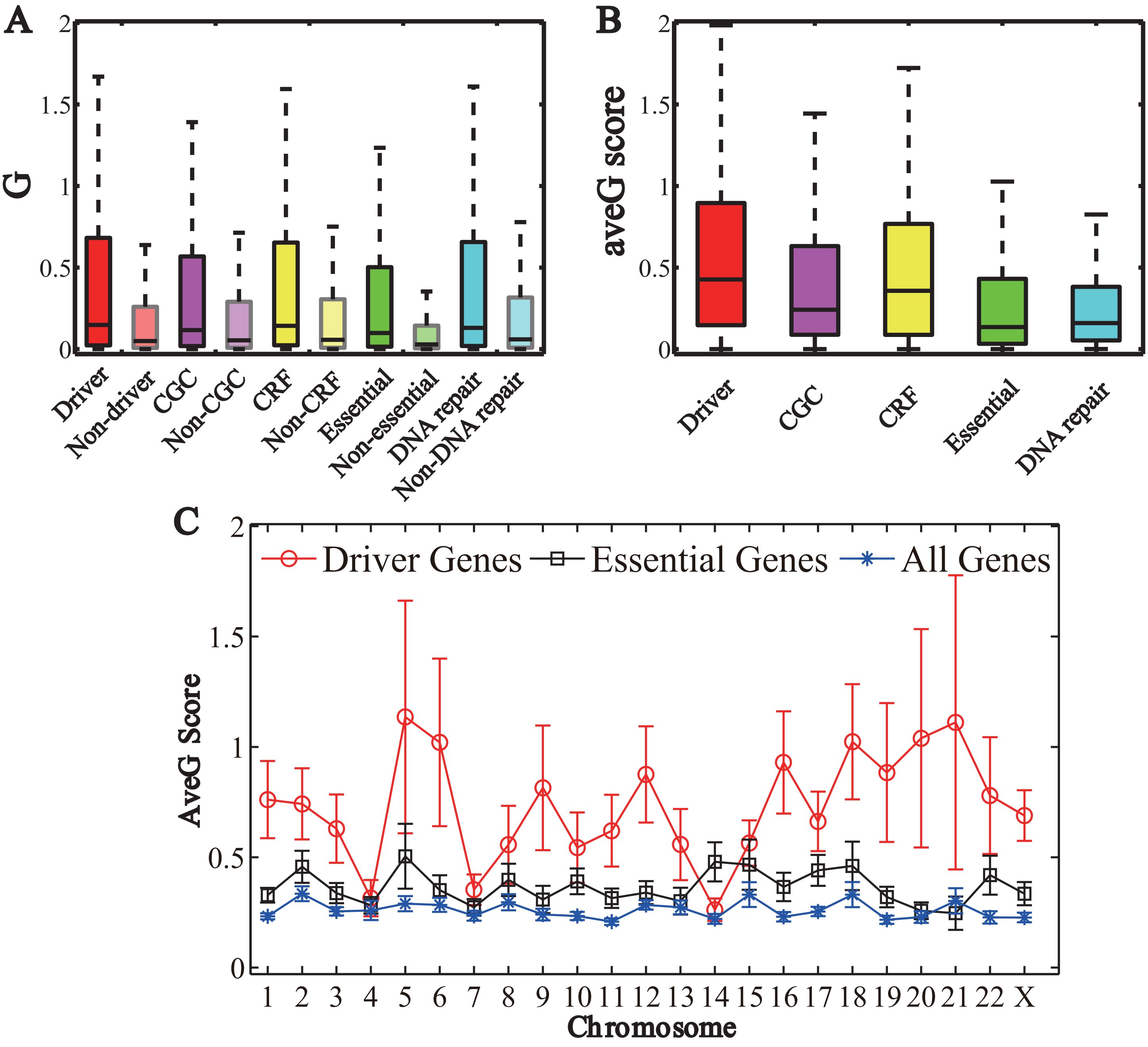

Fig. S23. The performance of the gene gravity model after removing 31 ultramutated tumor samples in colon adenocarcinoma (COAD). (**A**) Box plots of gene-gene gravitation score (G) distribution for five gene sets in COAD. Red: Cancer driver genes (driver) versus non-cancer driver genes (non-driver); purple: Cancer Gene Census (CGC) genes versus non-CGC genes; yellow: Chromatin regulation factors (CRFs) versus non-CRFs; green: Essential genes (Essential) versus non-essential genes (non-essential); blue: DNA repair genes versus non-DNA repair genes. (**B**) Box plots of gene average gravitation (aveG) score for five gene sets in COAD. (**C**) Distribution of average gravitation (aveG) score for cancer driver genes, essential genes, and all genes across 23 human chromosomes in COAD.
