# Supplementary material for: A Gene Gravity Model for the Evolution of Cancer Genomes: A Study of 3,000 Cancer Genomes across 9 Cancer Types
Source: PLoS Comput Biol. 2015 Sep 9;11(9):e1004497. doi: 10.1371/journal.pcbi.1004497 (PMC4564226; doi:10.1371/journal.pcbi.1004497)
Supplement: S26 Fig — (PDF) [file pcbi.1004497.s026.pdf]

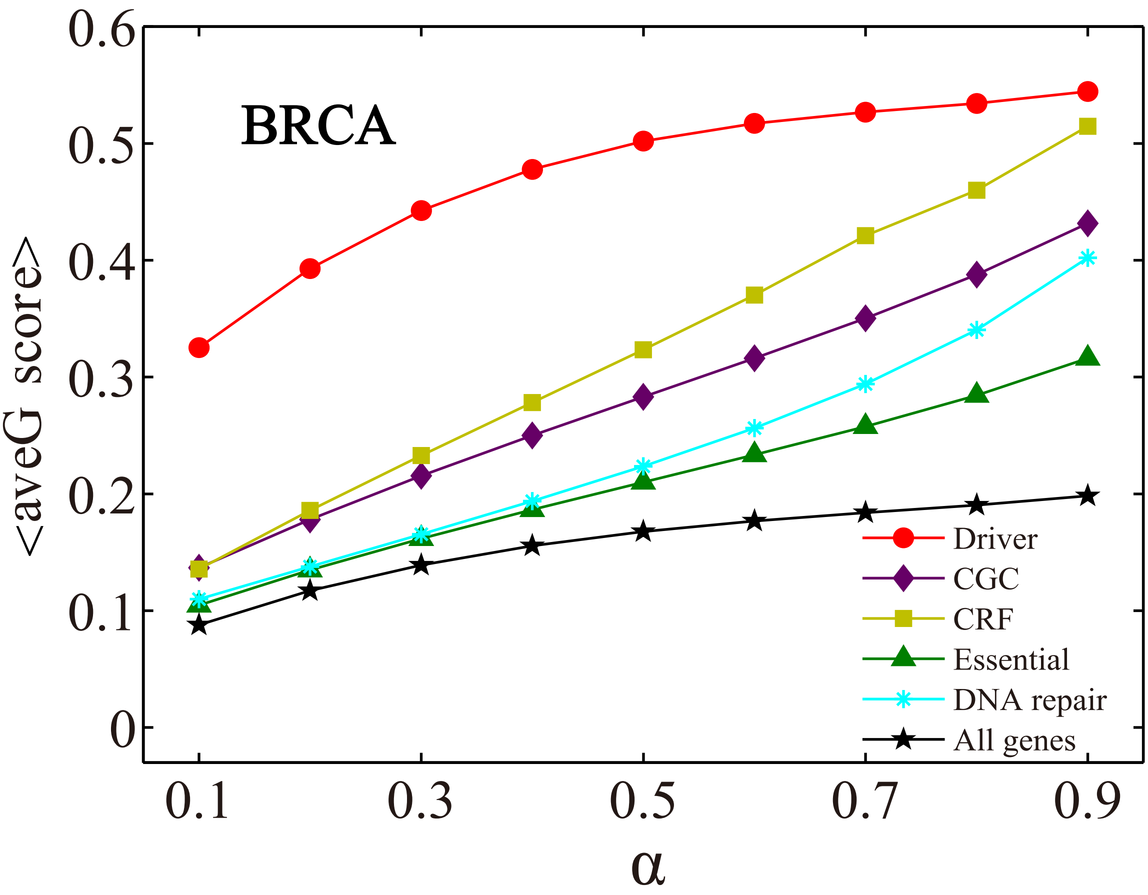

Fig. S26. The influence of gene average gravitation (aveG) scores changed by an important parameter Alpha during mutation network propagation in breast invasive carcinoma (BRCA). There is no propagation when α=0 and the mutation network propagation value of a gene is purely determined by the network structure when α=1. Red: Cancer driver genes (Driver); purple: Cancer Gene Census (CGC) genes; yellow: Chromatin regulation factors (CRF); green: Essential genes (Essential); blue: DNA repair genes, and black: all genes.
