# Supplementary material for: A Gene Gravity Model for the Evolution of Cancer Genomes: A Study of 3,000 Cancer Genomes across 9 Cancer Types
Source: PLoS Comput Biol. 2015 Sep 9;11(9):e1004497. doi: 10.1371/journal.pcbi.1004497 (PMC4564226; doi:10.1371/journal.pcbi.1004497)
Supplement: S27 Fig — (PDF) [file pcbi.1004497.s027.pdf]

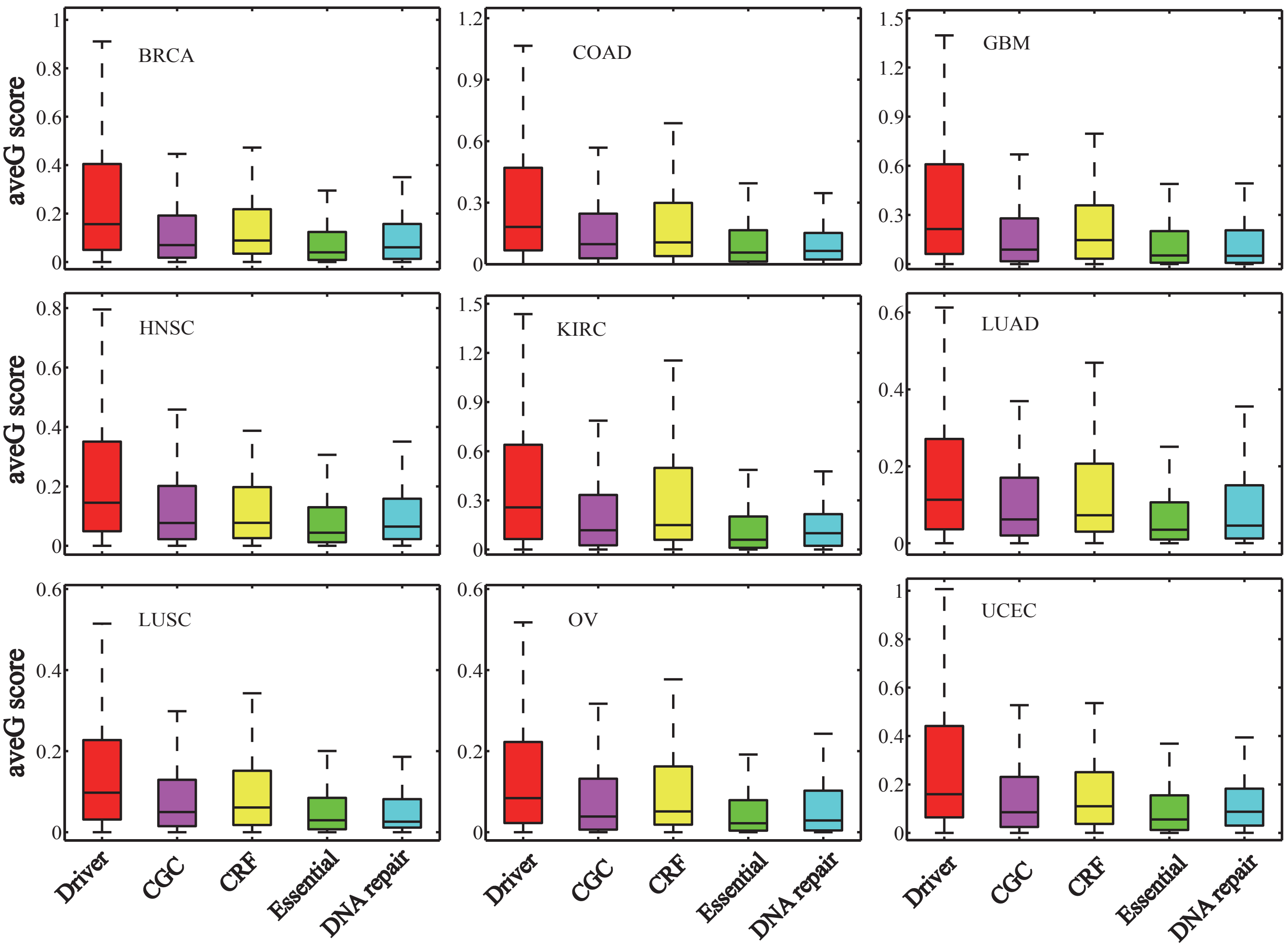

Fig. S27. Box plots of gene average gravitation (aveG) score for five gene sets across 9 cancer types when set up alpha = 0.2 during mutation network propagation. Red: Cancer driver genes (Driver); purple: Cancer Gene Census (CGC) genes; yellow: Chromatin regulation factors (CRF); green: Essential genes (Essential); and blue: DNA repair genes.
