# Supplementary material for: A Gene Gravity Model for the Evolution of Cancer Genomes: A Study of 3,000 Cancer Genomes across 9 Cancer Types
Source: PLoS Comput Biol. 2015 Sep 9;11(9):e1004497. doi: 10.1371/journal.pcbi.1004497 (PMC4564226; doi:10.1371/journal.pcbi.1004497)
Supplement: S1 Table — (PDF) [file pcbi.1004497.s028.pdf]

S1 Table. The statistics of transcriptional (RNA-seq) and somatic mutation profiles across 9 cancer types used in this study.

Cancer type	The number of samples (RNA-seq)	The number of samples (somatic mutations)	The number of somatic mutations
BRCA	988	771	31,408
LUAD	468	227	55,705
LUSC	482	177	43,898
KIRC	480	417	20,102
HNSC	303	305	39,714
COADREAD	232	213	28,012
UCEC	118	228	29,585
OV	262	316	13,815
GBM	154	291	15,131
Total	3,487	2,946	277,370

Abbreviations: LUAD: Lung adenocarcinoma, LUSC: lung squamous cell carcinoma, BRCA: breast adenocarcinoma, UCEC: uterine corpus endometrial carcinoma, COAD: colon and rectal carcinoma cancer, OV: ovarian serous carcinoma, GBM: glioblastoma multiforme, KIRC: kidney renal clear cell carcinoma, and HNSC: head and neck cancer.
