# Supplementary material for: A Gene Gravity Model for the Evolution of Cancer Genomes: A Study of 3,000 Cancer Genomes across 9 Cancer Types
Source: PLoS Comput Biol. 2015 Sep 9;11(9):e1004497. doi: 10.1371/journal.pcbi.1004497 (PMC4564226; doi:10.1371/journal.pcbi.1004497)
Supplement: S4 Table — (PDF) [file pcbi.1004497.s031.pdf]

S4 Table. The gene average gravitation score for 5 gene sets across 9 cancer types.

Cancer type	Average gravitation score ± standard deviation				
	Driver genes	CRFs	DNA repair genes	CGC genes	Essential genes
BRCA	0.47 ± 0.02	0.42 ± 0.04 (q=1.0)	0.30 ± 0.03 (q=1.9×10^{-4})	0.35 ± 0.02 (q=1.1×10^{-4})	0.26 ± 0.01 (q=2.3×10^{-32})
COAD	0.64 ± 0.04	0.55 ± 0.05 (q=1.0)	0.32 ± 0.04 (q=6.0×10^{-4})	0.36 ± 0.02 (q=3.9×10^{-3})	0.27 ± 0.01 (q=2.2×10^{-29})
GBM	0.82 ± 0.05	0.82 ± 0.07 (q=1.0)	0.51 ± 0.08 (q=9.0×10^{-6})	0.61 ± 0.04 (q=9.9×10^{-5})	0.46 ± 0.02 (q=4.6×10^{-31})
HNSC	0.46 ± 0.02	0.42 ± 0.04 (q =1.0)	0.32 ± 0.04 (q=6.0×10^{-4})	0.36 ± 0.02 (q=3.9×10^{-3})	0.27 ± 0.01 (q=8.7×10^{-68})
KIRC	0.82 ± 0.05	0.93 ± 0.13 (q=1.0)	0.47 ± 0.05 (q=1.8×10^{-4})	0.61 ± 0.05 (q=2.5×10^{-4})	0.41 ± 0.01 (q=1.7×10^{-31})
LUAD	0.42 ± 0.02	0.43 ± 0.04 (q=1.0)	0.32 ± 0.04 (q=3.0×10^{-3})	0.32 ± 0.02 (q=3.9×10^{-3})	0.25 ± 0.01 (q=2.2×10^{-29})
LUSC	0.33 ± 0.02	0.30 ± 0.03 (q=1.0)	0.21 ± 0.03 (q=1.6×10^{-5})	0.25 ± 0.02 (q=2.0×10^{-4})	0.20 ± 0.01 (q=4.4×10^{-30})
OV	0.30 ± 0.02	0.30 ± 0.03 (q=1.0)	0.18 ± 0.02 (q=2.0×10^{-5})	0.23 ± 0.01 (q=3.6×10^{-3})	0.17 ± 0.01 (q=4.8×10^{-30})
UCEC	0.58 ± 0.03	0.53 ± 0.06 (q=1.0)	0.38 ± 0.04 (q=4.8×10^{-3})	0.45 ± 0.03 (q=5.0×10^{-4})	0.30 ± 0.01 (q=1.4×10^{-30})

BRCA: breast invasive carcinoma, COAD: colon adenocarcinoma, GBM: glioblastoma multiforme, HNSC: head and neck squamous cell carcinoma, KIRC: kidney renal clear cell carcinoma, LUAD: lung adenocarcinoma, LUSC: lung squamous cell carcinoma, OV: ovarian serous cystadenocarcinoma, UCEC: uterine corpus endometrial carcinoma, CRF: chromatin regulation factors, and CGC: Cancer Gene Census. The p-values were calculated based on the comparison of the gene average gravitation score of cancer driver genes with CRFs, DNA repair genes, CGC genes, and essential genes, respectively, using Wilcoxon rank-sum test and corrected by Benjamini-Hochberg multiple testing.
