# Supplementary material for: A Gene Gravity Model for the Evolution of Cancer Genomes: A Study of 3,000 Cancer Genomes across 9 Cancer Types
Source: PLoS Comput Biol. 2015 Sep 9;11(9):e1004497. doi: 10.1371/journal.pcbi.1004497 (PMC4564226; doi:10.1371/journal.pcbi.1004497)
Supplement: S5 Table — (PDF) [file pcbi.1004497.s032.pdf]

S5 Table. The average gravitation score of CRF cancer driver genes, non-CRF cancer driver genes and non-driver CRF genes.

Cancer type	Average gravitation score ± standard deviation			Adjusted p-value (q)		
	CRF cancer driver genes	Non-CRF cancer driver genes	Non-driver CRF genes	**I**	**II**	**III**
BRCA	0.60 ± 0.10	0.46 ± 0.02	0.38 ± 0.05	0.18	0.06	1.0
COAD	0.77 ± 0.13	0.63 ± 0.04	0.50 ± 0.05	0.18	0.08	1.0
GBM	1.1 ± 0.17	0.80 ± 0.05	0.73 ± 0.08	0.08	0.06	1.0
HNSC	0.64 ± 0.12	0.44 ± 0.02	0.36 ± 0.04	0.16	0.06	1.0
KIRC	1.6 ± 0.48	0.76 ± 0.04	0.72 ± 0.09	4.2×10^{-3}	3.3×10^{-3}	1.0
LUAD	0.52 ± 0.07	0.41 ± 0.02	0.41 ± 0.04	0.08	0.08	1.0
LUSC	0.42 ± 0.06	0.32 ± 0.02	0.27 ± 0.03	0.10	0.04	1.0
OV	0.44 ± 0.08	0.29 ± 0.02	0.26 ± 0.02	0.08	0.08	1.0
UCEC	0.79 ± 0.21	0.56 ± 0.03	0.45 ± 0.05	0.18	0.08	1.0

The p-values in column **I** represent the Wilcoxon rank-sum test of average gravitation score between CRF cancer driver genes versus non-CRF cancer driver genes. The p-values in column **II** represent the Wilcoxon rank-sum test of average gravitation score between CRF cancer driver genes versus non-driver CRF genes. The p-values in column **III** represent the Wilcoxon rank-sum test of average gravitation score between non-CRF cancer driver genes and non-driver CRF genes. CRF: chromatin regulation factor. All p-values were corrected by Benjamini-Hochberg multiple testing in **S5-S12 Tables**.
