# Supplementary material for: A Gene Gravity Model for the Evolution of Cancer Genomes: A Study of 3,000 Cancer Genomes across 9 Cancer Types
Source: PLoS Comput Biol. 2015 Sep 9;11(9):e1004497. doi: 10.1371/journal.pcbi.1004497 (PMC4564226; doi:10.1371/journal.pcbi.1004497)
Supplement: S6 Table — (PDF) [file pcbi.1004497.s033.pdf]

S6 Table. The average gravitation score of CRF CGC genes, non-CRF CGC genes and non-CGC CRF genes.

Cancer type	Average gravitation score ± standard deviation			Adjusted p-value (*q*)		
	CRF CGC genes	Non-CRF CGC genes	Non-CGC CRF genes	**I**	**II**	**III**
BRCA	0.57 ± 0.13	0.33 ± 0.02	0.39 ± 0.04	0.03	0.46	0.08
COAD	0.70 ± 0.15	0.44 ± 0.03	0.52 ± 0.05	0.04	0.48	0.17
GBM	1.1 ± 0.20	0.58 ± 0.04	0.76 ± 0.08	7.0×10^{-4}	0.13	0.06
HNSC	0.65 ± 0.16	0.34 ± 0.02	0.37 ± 0.04	0.04	0.46	0.17
KIRC	1.9 ± 0.64	0.53 ± 0.04	0.74 ± 0.08	6.8×10^{-6}	6.2×10^{-3}	0.04
LUAD	0.56 ± 0.10	0.30 ± 0.02	0.41 ± 0.04	0.08	0.24	0.06
LUSC	0.39 ± 0.07	0.23 ± 0.02	0.29 ± 0.03	0.03	0.46	0.09
OV	0.36 ± 0.06	0.22 ± 0.01	0.29 ± 0.03	0.02	0.46	0.04
UCEC	0.82 ± 0.28	0.42 ± 0.03	0.47 ± 0.05	0.04	0.46	0.06

The p-values in column **I** represent the Wilcoxon rank-sum test of average gravitation score between CRF CGC genes versus non-CRF CGC genes. The p-values in column **II** represent the Wilcoxon rank-sum test of average gravitation score between CRF CGC genes versus non-CGC CRF genes. The p-values in column **III** represent the Wilcoxon rank-sum test of average gravitation score between non-CRF CGC genes and non-CGC CRF genes. CRF: chromatin regulation factor, CGC: cancer gene census.
