# Supplementary material for: A Gene Gravity Model for the Evolution of Cancer Genomes: A Study of 3,000 Cancer Genomes across 9 Cancer Types
Source: PLoS Comput Biol. 2015 Sep 9;11(9):e1004497. doi: 10.1371/journal.pcbi.1004497 (PMC4564226; doi:10.1371/journal.pcbi.1004497)
Supplement: S7 Table — (PDF) [file pcbi.1004497.s034.pdf]

S7 Table. The average gravitation score of DNA repair cancer driver genes, non-DNA repair cancer driver genes, and non-driver DNA repair genes.

Cancer type	Average gravitation score ± standard deviation			Adjusted p-value (q)		
	DNA repair cancer driver genes	Non-DNA repair cancer driver genes	Non-driver DNA repair genes	**I**	**II**	**III**
BRCA	0.73 ± 0.13	0.46 ± 0.02	0.24 ± 0.03	0.12	4.4×10^{-4}	3.8×10^{-6}
COAD	0.67 ± 0.11	0.64 ± 0.04	0.31 ± 0.04	0.15	3.2×10^{-4}	3.0×10^{-7}
GBM	1.2 ± 0.33	0.81 ± 0.05	0.43 ± 0.07	0.15	4.4×10^{-4}	1.5×10^{-7}
HNSC	0.44 ± 0.17	0.45 ± 0.02	0.25 ± 0.03	0.02	9.9×10^{-5}	6.9×10^{-6}
KIRC	1.2 ± 0.23	0.80 ± 0.05	0.38 ± 0.04	0.08	2.7×10^{-4}	1.8×10^{-6}
LUAD	0.68 ± 0.13	0.41 ± 0.02	0.28 ± 0.04	0.08	5.2×10^{-4}	1.7×10^{-4}
LUSC	0.41 ± 0.08	0.33 ± 0.02	0.18 ± 0.03	0.15	4.4×10^{-4}	3.0×10^{-7}
OV	0.43 ± 0.09	0.30 ± 0.02	0.15 ± 0.02	0.12	3.2×10^{-4}	3.0×10^{-7}
UCEC	0.70 ± 0.11	0.57 ± 0.03	0.34 ± 0.04	0.12	5.2×10^{-4}	5.5×10^{-4}

The p-values in column **I** represent the Wilcoxon rank-sum test of average gravitation score between DNA repair cancer driver genes and non-DNA repair cancer driver genes. The p-values in column **II** represent the Wilcoxon rank-sum test of average gravitation score between DNA repair cancer driver genes and non-driver DNA repair genes. The p-values in **III** represent the Wilcoxon rank-sum test of average gravitation score between non-DNA repair cancer driver genes and non-driver DNA repair genes.
