# Supplementary material for: A Gene Gravity Model for the Evolution of Cancer Genomes: A Study of 3,000 Cancer Genomes across 9 Cancer Types
Source: PLoS Comput Biol. 2015 Sep 9;11(9):e1004497. doi: 10.1371/journal.pcbi.1004497 (PMC4564226; doi:10.1371/journal.pcbi.1004497)
Supplement: S8 Table — (PDF) [file pcbi.1004497.s035.pdf]

S8 Table. The average gravitation score of DNA repair Cancer Gene Census (CGC) genes, non-DNA repair CGC genes, and non-CGC DNA repair genes.

Cancer type	Average gravitation score ± standard deviation			Adjusted p-value (q)		
	DNA repair CGC genes	Non-DNA repair CGC genes	Non-CGC DNA repair genes	**I**	**II**	**III**
BRCA	0.50 ± 0.10	0.34 ± 0.02	0.25 ± 0.03	0.68	0.12	0.20
COAD	0.46 ± 0.08	0.46 ± 0.03	0.32 ± 0.05	0.91	0.12	0.04
GBM	0.64 ± 0.12	0.61 ± 0.04	0.48 ± 0.09	0.91	0.12	0.09
HNSC	0.50 ± 0.12	0.35 ± 0.02	0.27 ± 0.04	0.91	0.25	0.17
KIRC	0.75 ± 0.16	0.60 ± 0.06	0.41 ± 0.05	0.91	0.12	0.16
LUAD	0.44 ± 0.08	0.31 ± 0.02	0.29 ± 0.04	0.68	0.12	0.19
LUSC	0.25 ± 0.05	0.24 ± 0.02	0.19 ± 0.03	0.91	0.12	0.11
OV	0.30 ± 0.06	0.22 ± 0.01	0.15 ± 0.02	0.77	0.03	0.02
UCEC	0.45 ± 0.07	0.45 ± 0.04	0.36 ± 0.04	0.68	0.12	0.64

The p-values in column **I** represent the Wilcoxon rank-sum test of average gravitation score between DNA repair CGC genes and non-DNA repair CGC genes. The p-values in column **II** represent the Wilcoxon rank-sum test of average gravitation score between DNA repair CGC genes and non-CGC DNA repair genes. The p-values in **III** represent the Wilcoxon rank-sum test of average gravitation score between non-DNA repair CGC genes and non-CGC DNA repair genes.
