# Supplementary material for: A Gene Gravity Model for the Evolution of Cancer Genomes: A Study of 3,000 Cancer Genomes across 9 Cancer Types
Source: PLoS Comput Biol. 2015 Sep 9;11(9):e1004497. doi: 10.1371/journal.pcbi.1004497 (PMC4564226; doi:10.1371/journal.pcbi.1004497)
Supplement: S9 Table — (PDF) [file pcbi.1004497.s036.pdf]

S9 Table. The enrichment analysis of the top 100 genes that have the highest gene average gravitation score between cancer driver genes and non-driver genes.

Cancer type	Number of driver genes	Number of non-driver genes	Adjusted p-value (q)	Odd ratio	Number of all driver genes	Number of all non-driver genes
BRCA	16	84	1.8×10^{-7}	6.3		
COAD	21	79	1.2×10^{-11}	8.9		
GBM	15	85	2.9×10^{-7}	5.8		
HNSC	17	83	3.4×10^{-8}	6.8		
KIRC	22	78	1.6×10^{-12}	9.4	614	19852
LUAD	18	82	5.6×10^{-9}	7.3		
LUSC	16	84	1.8×10^{-7}	6.3		
OV	16	84	1.8×10^{-7}	6.3		
UCEC	22	78	1.6×10^{-12}	9.4		
