# Supplementary material for: A Gene Gravity Model for the Evolution of Cancer Genomes: A Study of 3,000 Cancer Genomes across 9 Cancer Types
Source: PLoS Comput Biol. 2015 Sep 9;11(9):e1004497. doi: 10.1371/journal.pcbi.1004497 (PMC4564226; doi:10.1371/journal.pcbi.1004497)
Supplement: S10 Table — (PDF) [file pcbi.1004497.s037.pdf]

S10 Table. The enrichment analysis of the top 100 genes that have the highest gene average gravitation score between cancer gene census (CGC) and non-CGC.

Cancer type	Number of CRF genes	Number of non-CRF genes	Adjusted p-value (*q*)	Odd ratio	Number of CRF genes	Number of all non-CRF genes
BRCA	9	91	4.5×10^{-3}	4.1		
COAD	8	92	0.01	3.6		
GBM	9	91	4.5×10^{-3}	4.1		
HNSC	8	92	0.01	3.6		
KIRC	9	91	4.5×10^{-3}	4.1	487	19980
LUAD	6	94	0.03	2.6		
LUSC	10	90	1.1×10^{-3}	4.6		
OV	8	92	0.01	3.6		
UCEC	12	88	4.1×10^{-5}	5.7		
