# Supplementary material for: A Gene Gravity Model for the Evolution of Cancer Genomes: A Study of 3,000 Cancer Genomes across 9 Cancer Types
Source: PLoS Comput Biol. 2015 Sep 9;11(9):e1004497. doi: 10.1371/journal.pcbi.1004497 (PMC4564226; doi:10.1371/journal.pcbi.1004497)
Supplement: S11 Table — (PDF) [file pcbi.1004497.s038.pdf]

S11 Table. The enrichment analysis of the top 100 genes that have the highest gene average gravitation score between chromatin regulation factors (CRFs) and non-CRFs.

Cancer type	Number of CRF genes	Number of non-CRF genes	Adjusted p-value (*q*)	Odd ratio	Number of CRF genes	Number of all non-CRF genes
BRCA	6	94	1.6×10^{-3}	7.6		
COAD	3	97	0.12	3.6		
GBM	4	96	0.04	4.9		
HNSC	4	96	0.04	4.9		
KIRC	7	93	2.3×10^{-4}	9.0	176	20286
LUAD	7	93	2.3×10^{-5}	9.0		
LUSC	3	97	0.12	3.6		
OV	6	94	1.6×10^{-3}	7.6		
UCEC	5	95	8.5×10^{-3}	6.2		
