# Supplementary material for: A Gene Gravity Model for the Evolution of Cancer Genomes: A Study of 3,000 Cancer Genomes across 9 Cancer Types
Source: PLoS Comput Biol. 2015 Sep 9;11(9):e1004497. doi: 10.1371/journal.pcbi.1004497 (PMC4564226; doi:10.1371/journal.pcbi.1004497)
Supplement: S12 Table — (PDF) [file pcbi.1004497.s039.pdf]

S12 Table. The enrichment analysis of the top 100 genes that have the highest gene average gravitation score between DNA repair genes and non-DNA repair genes.

Cancer type	Number of DNA repair genes	Number of non-DNA repair genes	Adjusted p-value (*q*)	Odd ratio	Number of all DNA repair genes	Number of all non- DNA repair genes
BRCA	2	98	1.0	2.7		
COAD	1	99	1.0	1.3		
GBM	3	97	0.32	4.2		
HNSC	4	94	0.06	5.8		
KIRC	2	98	1.0	2.7	153	20310
LUAD	2	98	1.0	2.7		
LUSC	1	99	1.0	1.3		
OV	2	98	1.0	2.7		
UCEC	1	99	1.0	1.3		

Note: p-value in **S9-S12 Tables** was calculated using Fisher' exact test, and was corrected as adjusted p-values (*q*) by Benjamini-Hochberg multiple testing.
